# Global insights on flood risk mitigation in arid regions using geomorphological and geophysical modeling from a local case study

**DOI:** 10.1038/s41598-024-69541-x

**Published:** 2024-08-28

**Authors:** Adel Kotb, Ayman I. Taha, Ahmed A. Elnazer, Alhussein Adham Basheer

**Affiliations:** 1 Geology Department, Faculty of Science, Helwan University, Ain Helwan, Cairo, 11795 Egypt; 2 Geoelectric and Geomagnetism Department, National Research Institute of Astronomy and Geophysics (NRIAG), Helwan, Cairo, 11421 Egypt; 3 Geological Sciences Department, National Research Centre (NRC), 33 El Bohouth St. (Former El-Tahrir St.), Dokki, Giza, 12622 Egypt

**Keywords:** Mitigating the flood danger, Arid regions, Geomorphological analysis, Geophysical modeling, Hydrological assessment, Dam site selection, Wadi Al-Laith, Saudi Arabia, Environmental sciences, Natural hazards

## Abstract

This research provides a comprehensive examination of flood risk mitigation in Saudi Arabia, with a focus on Wadi Al-Laith. It highlights the critical importance of addressing flood risks in arid regions, given their profound impact on communities, infrastructure, and the economy. Analysis of morphometric parameters ((drainage density (Dd), stream frequency (Fs), drainage intensity (Di), and infiltration number (If)) reveals a complex hydrological landscape, indicating elevated flood risk. due to low drainage density, low stream frequency, high bifurcation ratio, and low infiltration number. Effective mitigation strategies are imperative to protect both communities and infrastructure in Wadi Al-Laith. Geophysical investigations, using specialized software, improve the quality of the dataset by addressing irregularities in field data. A multi-layer geoelectric model, derived from vertical electrical sounding (VES) and time domain electromagnetic (TDEM) surveys, provides precise information about the geoelectric strata parameters such as electrical resistivity, layer thicknesses, and depths in the study area. This identifies a well-saturated sedimentary layer and a cracked rocky layer containing water content. The second region, proposed for a new dam, scores significantly higher at 56% in suitability compared to the first region’s 44%. The study advocates for the construction of a supporting dam in the second region with a height between 230 and 280 m and 800 m in length. This new dam can play a crucial role in mitigating flash flood risks, considering various design parameters. This research contributes to flood risk management in Saudi Arabia by offering innovative dam site selection approaches. It provides insights for policymakers, researchers, and practitioners involved in flood risk reduction, water resource management, and sustainable development in arid regions globally.

## Introduction

Floods have long been recognized as one of the most devastating natural disasters, posing significant threats to communities worldwide^1^. In the Kingdom of Saudi Arabia (KSA), a region characterized by arid landscapes and sporadic rainfall, floods can have catastrophic consequences^2^. This paper aims to address the multifaceted issue of flood risks and their profound impact on the communities of KSA^3,4^. Furthermore, it underscores the critical importance of mitigating these risks through the judicious selection of dam sites, emphasizing the utilization of geophysical and geomorphological modeling techniques^5^.

Floods in KSA, while infrequent, are nonetheless devastating when they occur due to the arid nature of the region^6^. These events can lead to loss of life, damage to infrastructure, disruption of livelihoods, and economic losses^7^. Understanding the dynamics of flood risks is essential for safeguarding the well-being of KSA’s communities and ensuring the sustainable development of the region^8^. One pivotal approach to mitigating the impact of floods in KSA is through the strategic placement of dams^9^. These structures play a vital role in flood control, water resource management, and supporting agricultural activities^10^. Therefore, the selection of appropriate dam sites is paramount to the overall flood risk reduction strategy.

**Figure 1:**
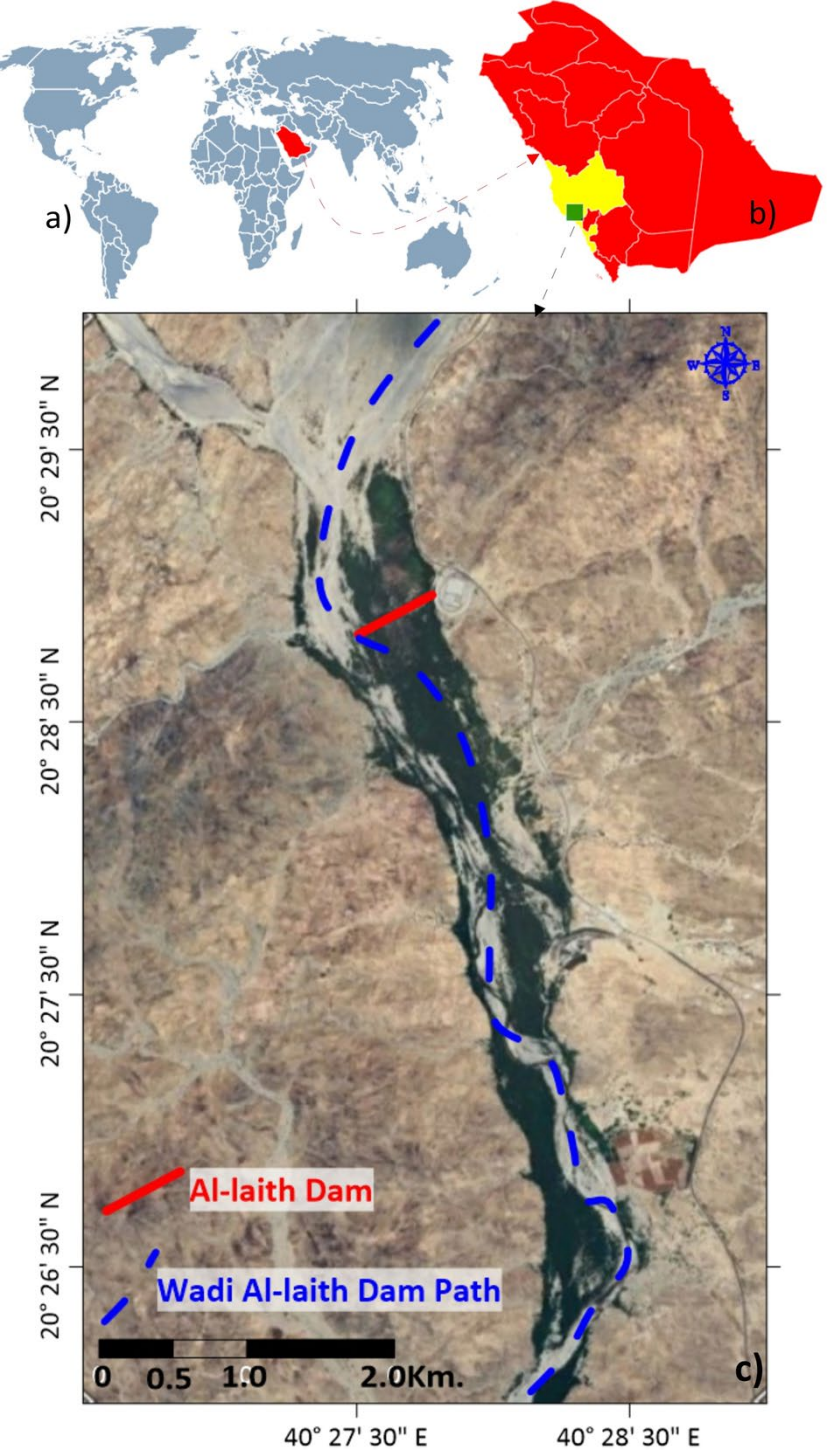
location map of the study area, (**a**) spatial location of Saudi Arabia (red) relative to the world (gray) created by map chart, https://www.mapchart.net/world.html, (**b**) spatial location of Wadi Laith (green) relative to Makkah governorate (yellow) in Saudi Arabia created by map chart, https://www.mapchart.net/asia.html, (**c**) spatial location of Wadi Laith [created using^26^.

In this context, this paper centers its focus on the application of geophysical and geomorphological modeling techniques, specifically within the unique setting of Wadi Al-Laith in KSA^11^. Wadi Al-Laith, characterized by its intricate topography and hydrological features, serves as an exemplary case study to demonstrate the efficacy of these innovative approaches in dam site selection (Fig. 1). To contextualize our research, we present a comprehensive review of previous studies related to flood risks and dam site selection within the KSA region^12^. These studies provide valuable insights into the historical context and existing methodologies employed in flood risk management. Acknowledging the limitations and challenges of existing approaches is fundamental to driving innovation in flood risk mitigation^13^. By critically evaluating past strategies, we can identify areas where geophysical and geomorphological modeling can enhance the accuracy and effectiveness of dam site selection^14,15^.

This study’s objective is toestablish a holistic framework for enhancing flood risk mitigation strategies in the region and contribute to the ongoing discourse on flood risk management in KSA by exploring innovative approaches to dam site selection, particularly through a promising solution of the application of geophysical and geomorphological modeling^16,17^. It endeavors to offer recommendations that can advance the planning and selection of optimal locations for new dams, as well as evaluate the performance and efficiency of existing dams^18,19^. Ultimately, this research is dedicated to ensuring the protection of both the communities and critical infrastructure within the Kingdom of Saudi Arabia (KSA).

The primary aim of this paper is to comprehensively address the multifaceted issue of flood risks in the Kingdom of Saudi Arabia (KSA), highlighting the unique challenges posed by the region’s arid landscapes and sporadic rainfall. The paper emphasizes the catastrophic consequences floods can have on communities, infrastructure, livelihoods, and the economy within KSA, while also considering global implications. It underscores the critical importance of mitigating flood risks through the judicious selection of dam sites, advocating for the use of advanced geophysical and geomorphological modeling techniques to enhance decision-making processes. Recognizing the devastating impact of floods in KSA despite their infrequency, the study promotes the strategic placement of dams as essential for flood control, sustainable water resource management, and supporting agricultural activities.

The paper specifically focuses on Wadi Al-Laith as a case study to illustrate the efficacy of geophysical and geomorphological modeling techniques in dam site selection. This area’s intricate topography and hydrological features serve as an exemplary setting for showcasing innovative flood risk mitigation strategies that could potentially inform similar efforts globally. To provide a comprehensive context, the paper conducts a thorough review of previous studies related to flood risks and dam site selection within KSA, aiming to offer insights into historical contexts, existing methodologies, and challenges faced in flood risk management.

Moreover, the study aims to contribute to the global discourse on flood risk management by exploring innovative approaches to dam site selection that improve accuracy and effectiveness through advanced modeling techniques. By establishing a holistic framework for enhancing flood risk mitigation strategies in KSA, the paper seeks to provide recommendations that can advance the planning and selection of optimal dam locations and evaluate the performance of existing infrastructure. Ultimately, the research aims to protect communities and critical infrastructure in KSA and beyond, thereby improving global resilience to floods and promoting sustainable development practices worldwide.

## Area under investigation

Wadi Al-Laith, located in the Kingdom of Saudi Arabia (KSA), is a distinctive geographical feature within the western region of the country^20^. It is characterized by a variety of unique geographical attributes that shape its landscape and hydrology (Fig. 1).

Wadi Al-Laith can be described as a wadi, which is a typically dry riverbed or valley that experiences sporadic and often intense flash floods during the rare rainfall events in the arid region of KSA^21^. The geographical features of Wadi Al-Laith include a meandering topography with a pronounced channel that can expand dramatically during flood events. The valley exhibits a narrow and winding path, surrounded by rocky terrain and outcrops, with the nearby presence of limestone formations^22^.

The region’s hydrology is further influenced by its proximity to the Red Sea and the surrounding mountain ranges, which can contribute to localized weather patterns and rainfall variability^23^. Due to its geological composition and topographical characteristics, Wadi Al-Laith becomes particularly susceptible to flash flooding, making it a pertinent area for studying flood risk mitigation.

Wadi Al-Laith has witnessed several historical flood events, which have had significant repercussions for the surrounding communities and infrastructure^14,24^. These flood events are typically associated with the sporadic but intense rainstorms that occasionally occur in the region. Over the years, these floods have resulted in loss of life, damage to property, disruption of transportation networks, and agricultural losses. These historical flood events serve as poignant reminders of the urgent need to develop effective flood risk mitigation strategies in the area^20,21,25^.

Wadi Al-Laith assumes paramount importance as a case study for flood risk mitigation and dam site selection for several compelling reasons. Firstly, the unique topographical and geological characteristics of the region, such as the presence of limestone formations and rocky outcrops, make it an ideal testing ground for assessing the effectiveness of mitigation measures, including the strategic placement of dams. Secondly, the historical flood events in Wadi Al-Laith provide valuable data and insights into the vulnerabilities and risks associated with flash floods in arid regions, which can inform the development of targeted mitigation strategies. Thirdly, the lessons learned from Wadi Al-Laith can be extrapolated to other wadis and flood-prone areas within KSA and similar arid regions globally, making it a crucial reference point for policymakers, researchers, and practitioners engaged in flood risk management.

Briefly, Wadi Al-Laith in KSA serves as an exemplary study area for comprehensively examining the geographical characteristics, historical flood events, and the imperative role it plays in advancing flood risk mitigation and dam site selection strategies. The insights gained from this case study have the potential to enhance the resilience of communities and infrastructure in arid regions, safeguarding them against the adverse impacts of flash floods.

## Geological settings

**Figure 2:**
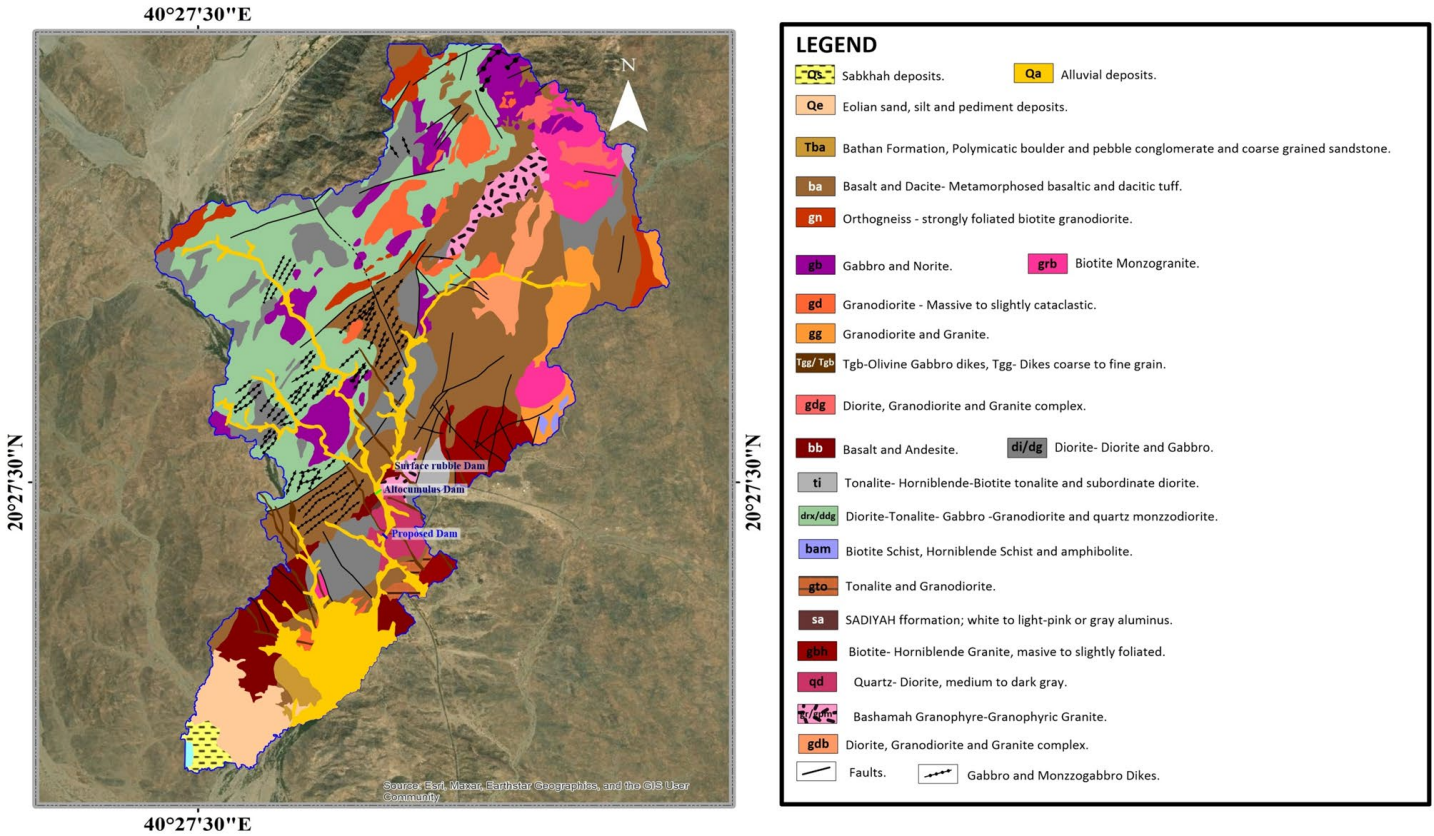
Geological map of the area under investigation and its surroundings. [Created using^34^.

Wadi Al Lith, situated in the western region of Saudi Arabia, boasts a distinctive geological landscape characterized by its diverse features (Fig. 2). The prevailing geological composition of this area primarily consists of sedimentary rocks, prominently marked by the presence of extensive limestone formations^27^. These limestone formations are integral components of the sedimentary sequence affiliated with the Arabian Platform, with origins traceable to the Cretaceous and Paleogene epochs^26,28^. Specifically, the study area within Wadi Al-Lith assumes the form of a valley stream typified by a thin sedimentary layer, the close proximity of hard rock strata to the surface, and rocky outcrops flanking the valley’s margins^29^. Notably, in select regions, sediment thickness within the valley gradually increases until it interfaces with the underlying hard rock formations.

The geological framework of the Wadi Al-Lith catchment area comprises four primary rock units, as detailed by^27,30^.

I. Quaternary, encompassing sand, gravel, and silt deposits: yhis unit exhibits the predominant presence of eolian sand-dune formations and sheet sand and silt deposits, with sand deposits covering a substantial portion of the region.
II. Late- to post-tectonic granitic rocks: represented by various plutonic rock types, including diorite, tonalite, granodiorite, and monzogranite, alongside serpentinite to syenite formations.
III. Lith suite, Khasrah complex, diorite, and gabbro: constituting a suite of mafic to intermediate plutonic rocks.
IV. Baish and Baha groups: comprising rocks such as basalt–dacite and biotite-hornblende-schist-amphibolite.

Additionally, Wadi Al-Lith encompasses volcanic rocks, notably basalt and andesite, remnants of ancient volcanic activity^2^. These volcanic formations are associated with the Red Sea rift system, a significant geological phenomenon that has profoundly influenced the region’s topographical characteristics^31^.

Structurally, the geology of the Wadi Al-Lith region is shaped by faulting and folding processes. Underlying the sedimentary rocks is the Arabian Shield, a Precambrian-age basement complex^31^. Characterized by its rugged and mountainous terrain, this geological foundation contributes significantly to the diverse topography evident in the area^27,32^.

The presence of a multitude of rock types and geological structures within Wadi Al-Lith holds significant implications for water resources and the occurrence of flash floods. Impermeable rock formations, such as limestone, can expedite surface runoff during intense precipitation events, augmenting the susceptibility to flash floods^27,32^. Consequently, a profound comprehension of the geological attributes of the region assumes paramount importance in facilitating effective water resource management and the implementation of appropriate mitigation measures aimed at mitigating the impact of flash floods^31,32^.

## Methodology

The hydrogeological method in this study primarily involves using hydrological models to predict and map regions prone to flash floods. The geophysical methods employed include electrical resistivity sounding (VES) and time-domain electromagnetic (TDEM) methods to investigate subsurface layers. Combining hydrogeological and geophysical methods offers a comprehensive understanding of the factors influencing flash floods. Hydrological models derived from detailed morphometric and land cover analyses are augmented with subsurface information obtained from geophysical measurements. This integrated approach allows for more accurate predictions of flash flood-prone areas by considering both surface characteristics and subsurface conditions, ultimately enhancing flood risk mitigation strategies.

### Hydrogeological method

In this study, hydrological models assume a pivotal role in the anticipation and mapping of flash flood-prone regions. The hydrological models used in this study are advanced and multifaceted, incorporating: (a) morphometric analysis which are utilizing parameters like drainage density, stream frequency, and rainage intensity, (b) topographic data derived from high-resolution topographic maps, (c) land cover data (integrated using the ASTER GDEM dataset), (d) subsurface information (enhanced with data from geophysical methods), and e) GIS Software: ArcGIS 10.4.1(for comprehensive data analysis).

These models work together to predict specific locales susceptible to flash floods, considering both surface and subsurface characteristics, to provide a holistic approach to flood risk mitigation in arid regions like the Kingdom of Saudi Arabia. These models find their genesis in morphometric analyses, which entail a comprehensive examination of the terrain's spatial characteristics and configurations. Topographic maps, boasting a horizontal posting resolution of approximately 30 m at the equatorial belt, serve as the primary data source for these morphometric inquiries. This level of detail facilitates an exhaustive comprehension of the landscape’s morphology and its ensuing influence on the hydrological patterns governing water flow.

To bolster the precision of the hydrological models, supplementary data regarding land cover is incorporated into the analytical framework. The research team leverages the ASTER Global Digital Elevation Model (GDEM) Version 3^33^, a dataset that furnishes a worldwide digital elevation model of terrestrial regions. This dataset boasts a spatial resolution of 1 arcsecond, equating to approximately 30 m on the ground. By integrating this land cover information into the hydrological models, the research endeavor accommodates pertinent factors such as vegetation types, soil compositions, and land use patterns, all of which exert substantial influences on the hydrological dynamics across the landscape.

Subsequently, hydrological models are brought into action to predict the specific locales susceptible to flash floods. These models simulate the water’s flow trajectory predicated on the amalgamation of topographic particulars and land cover attributes. In so doing, these models pinpoint areas where the confluence of terrain features and land cover characteristics renders them predisposed to the occurrence of flash floods. To further bolster the predictive capacity of these models, subsurface information procured through geophysical measurements is incorporated.

For the comprehensive analysis of data, including morphometric assessments, ArcGIS 10.4.1 software^34^ is employed. This software platform facilitates data visualization, manipulation, and morphometric analyses, enabling a detailed exploration of the study area’s pertinent parameters. Key morphometric parameters essential to this study are presented in Table 1, encompassing metrics such as drainage density (Dd), stream frequency (Fs), drainage intensity (Di), and infiltration number (If). These parameters, as outlined by^35,36^, form the cornerstone of the morphometric analyses undertaken in this investigation.

**Table 1:** Morphometric parameters for the study area.

Parameter	Formula	Reference
Drainage density (Dd)	Dd = Lu/A	^35^
Stream frequency (Fs)	Fs = Nu/A	^36^
Drainage intensity (Di)	Di = Fs/Dd	^36^

### Geophysical methods

**Figure 3:**
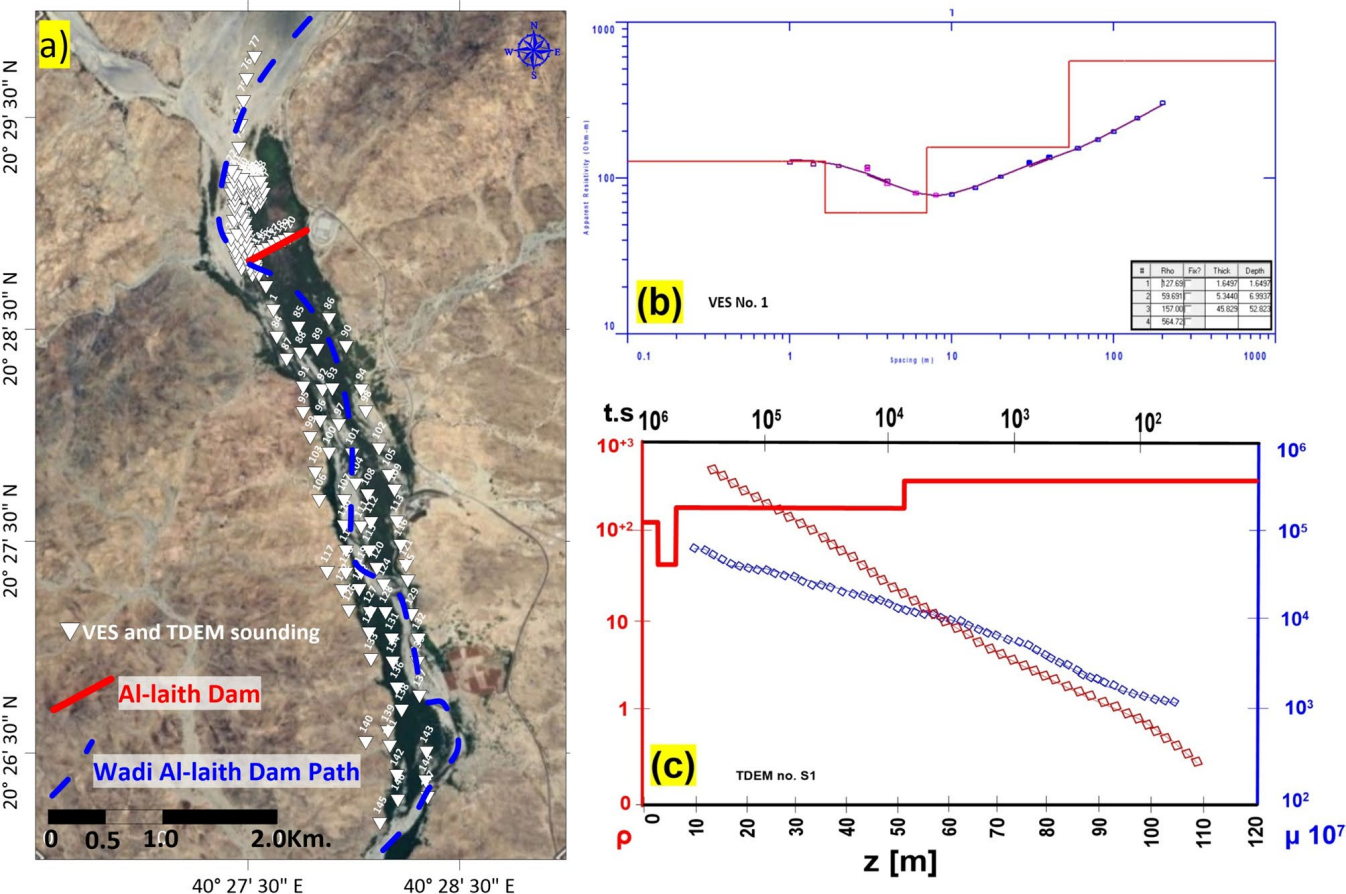
(**a**) Geographical distribution of VES and TDEM soundings’ site in the area under investigation [created using^26^, (**b**) example of VES no. 1 interpretation [extracted from^51^, (**c**) example of TDEM sounding no. 1 interpretation [extracted from^52^.

Geophysical methods, including electrical resistivity sounding (VES) and time-domain electromagnetic (TDEM) methods, are employed to investigate subsurface layers. The number of measurements were 157 VES and the same number of TDEM have been conducted in the same place to cover the whole area under investigation (Fig. 3a). VES measures subsurface electrical resistivity at various points, yielding insights into subsurface composition and properties. In contrast, TDEM employs electromagnetic pulses to assess subsurface characteristics. These geophysical measurements inform the development of subsurface models.

#### Geoelectrical method

Geoelectrical surveys, also known as the “DC method,” entail injecting direct electric current into the ground using surface-based current and voltage electrodes. The current’s direction is alternated to mitigate natural ground interference.

The vertical electrical sounding (VES) technique, utilizing continuous direct current (DC), is widely employed for groundwater exploration. It gauges values influenced by water content in rocks; higher values are characteristic of unsaturated rocks, while lower values indicate saturation, with salinity influencing measurements^37^.

The method of measuring ground electrical resistance relies primarily on Ohm's law, which states that the electric current flowing through a conductor is directly proportional to the voltage across it Eq. (1).

1 $${\text{V}} = {\text{IR}}$$

Ground electrical resistance is measured in accordance with Ohm’s law, where electric current is injected into the ground via two conductive electrodes (A and B)^38,39^Eqs. (2), (3).

2 $$\rho {\text{a }} = {\text{ K }}*\Delta {\text{V}}/{\text{I}}$$

3 $${\text{K }} = { 2}\pi /\left( {{1}/{\text{AM}} - {1}/{\text{AN}} - {1}/{\text{BM}} + {1}/{\text{BN}}} \right)$$

The apparent electrical resistance (ρa) is determined by dividing the product of the potential difference (∆V) by the current strength (I) and multiplying it by a geometric constant (K), which varies based on the distance between the current and voltage electrodes. This process is conducted using the Schlumberger configuration, which allows for deeper measurements compared to other configurations^40,41^.

Simultaneously, the potential difference across two additional electrodes (M and N) within the ground is measured. Apparent electrical resistance (ρa) is calculated by dividing the product of potential difference (∆V) by current strength (I) and multiplying by a geometric constant (K), contingent on the electrode distance. The Schlumberger configuration is employed for deeper measurements Eqs. (2),(3)^42^.

The geoelectrical survey in the study area was performed using the ARES II/1^43^ device, manufactured in the Czech Republic, which has a high capacity to transmit a current of up to 5 A, a voltage of 2000 V, and a capacity of up to 850 W, enabling measurements to be taken until reaching the solid base rocks.

#### Time domain electromagnetic method (TDEM)

TDEM relies on electromagnetic induction principles, creating a varying magnetic field and measuring induced electrical currents in the subsurface.

A transmitter coil carrying a strong current generates a changing magnetic field penetrating the subsurface. This field induces secondary electrical currents (eddy currents) in conductive materials beneath the surface, resulting in secondary magnetic fields. Upon deactivating the transmitter coil, the eddy currents decay, and the associated magnetic fields diminish. A receiver coil captures changes in the magnetic field over time, known as the decay curve or decaying electromagnetic response, providing subsurface resistivity distribution insights^44^.

Key equations utilized in TDEM include Faraday’s law of electromagnetic induction, Maxwell’s equations Eq. (4), governing electromagnetic wave propagation, and Ampere’s law, accounting for electric currents and the displacement current.

4 $$\nabla \times {\text{ B }} = {\upmu }_{0} {\text{J }} + {\upmu }_{0} {\upvarepsilon }_{0} {{\partial {\text{E}}} \mathord{\left/ {\vphantom {{\partial {\text{E}}} {\partial {\text{t}}}}} \right. \kern-0pt} {\partial {\text{t}}}}$$

where (∇ × B) is the curl of the magnetic field vector (B), (μ_0_) is the permeability of free space, a fundamental constant, (J) is the electric current density, and (∂E/∂t) is the rate of change of the electric field vector (E) with respect to time. This equation relates magnetic fields to electric currents and the displacement current (the term involving ∂E/∂t), which accounts for the changing electric field inducing a magnetic field^45,46^.

The Cole–Cole model represents complex electrical conductivity in subsurface materials, incorporating parameters (σʹ, σʹʹ, and α) to account for frequency-dependent conductivity Eq. (5).

5 $$\sigma^{*} = \sigma^{\prime } - {\text{j}}\sigma^{\prime \prime }$$

where the complex conductivity (σ*) and angular frequency (ω) and (j) is the imaginary unit (√(− 1))^40,41^.

Inversion algorithms, based on forward modeling and optimization techniques, interpret TDEM data and construct subsurface resistivity models. The inversion process involves comparing predicted data with measured data and adjusting the resistivity model to minimize discrepancies. Iterations continue until a satisfactory match is achieved, yielding the best-fitting resistivity distribution. These methodologies enable the estimation of subsurface properties, valuable in groundwater exploration, mineral assessment, and geological formation characterization^47^. Figure 3b, c illustrates an example of these interpretations.

By combining hydrological models derived from topographic and land cover data with the subsurface model obtained from geophysical measurements, a comprehensive understanding of the factors affecting the occurrence of flash floods can be achieved. This integrated approach allows for more accurate prediction of locations vulnerable to flash floods, as it takes into account surface characteristics and subsurface conditions.

In a clearer and more summarized sense, the hydrological models used in this study are derived from detailed morphometric studies based on topographic maps and land cover data. ASTER’s Global Digital Elevation Model (GDEM) version 3 is used to obtain land cover information. These models, along with subsurface information obtained through geophysical measurements and interpretation using VES and TDEM methods, contribute to predicting locations vulnerable to flash floods through a more comprehensive and accurate understanding of the contributing factors.

## Results

### Hydrogeological modeling

In this study, a comprehensive analysis of the study area’s topography, hydrology, and precipitation patterns was conducted using various geospatial data sources and techniques. The digital elevation model (DEM) played a central role in extracting valuable insights.

**Figure 4:**
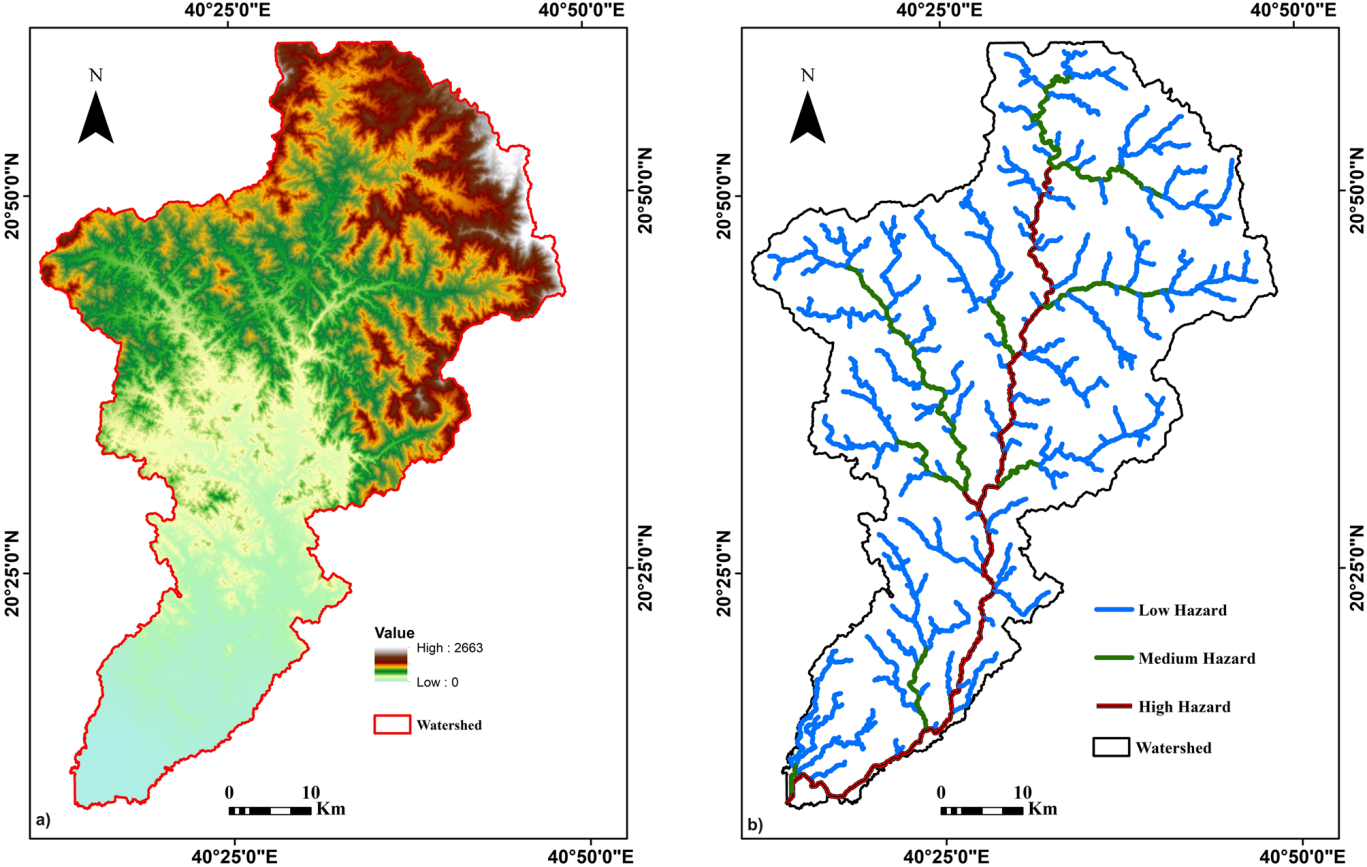
(**a**) Digital elevation map of the area under investigation, (**b**) drainage network map of the area under investigation. Created using^34^.

The DEM was employed to delineate the drainage network within the study area, specifically focusing on the Wadi Lith watershed (Fig. 4a). By assessing stream orders within this watershed, a significant observation emerged. It was noted that as the stream order increased, the number of associated stream segments decreased. Notably, the first-order stream (SU1) displayed the highest frequency, indicating that lower-order streams are more prevalent in the area. This observation underscores the heightened susceptibility of Wadi Lith to drainage-related hazards (Fig. 4b).

The DEM dataset yielded critical information concerning the topography and hydrology of the study area. Elevation data, flood flow directions, and identification of vulnerable regions were among the key findings derived from the DEM analysis. The elevation levels captured by the DEM ranged from 0 to 2663 m within the study area (Fig. 4a).

**Figure 5:**
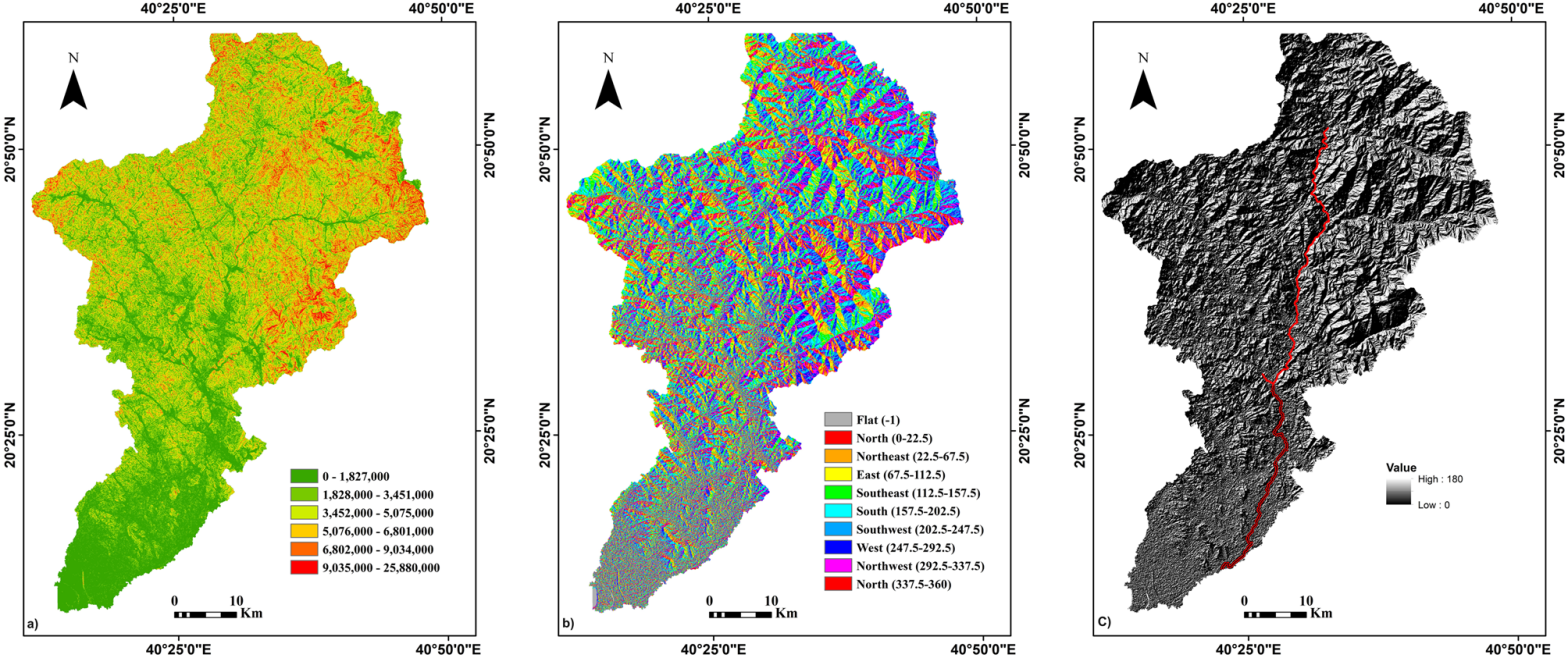
(**a**) Slope map of the area under investigation, (**b**) aspect map of the area under investigation, (**c**) Hill shade map of the area under investigation. [created using^34^.

The researchers employed ArcGIS software to generate three essential maps using the DEM data: slope, aspect, and hill shade maps to gain a deeper understanding of the topographic features. These maps provided distinct perspectives on the terrain’s characteristics. The slope map (Fig. 5a) vividly illustrated the steepness of the rocks in the study area, with higher slope values indicating more pronounced inclinations. The aspect map (Fig. 5b) revealed that slopes predominantly faced southward within the study area. Furthermore, the hill shade map (Fig. 5c), employing shading techniques, effectively portrayed the topographical features of hills and mountains. It accentuated relative slopes and mountain ridges, notably highlighting the valley of Al-Lith as particularly susceptible to flood hazards (Table 2).

**Table 2:** Some parameters of the studied Basin.

	Stream order (SU)							Total stream number (Nu)	Mean bifurcation ratio (Mbr)
	1	2	3	4	5	6	7		
Stream number (Nu)	4827	2409	1118	613	347	187	86	9587	–
Bifurcation ratio (Rb)	2.00	2.15	1.82	1.77	1.86	2.17	–		1.96

Monthly precipitation data (Table 3) were scrutinized to understand the precipitation patterns in the Al-Lith area. The analysis revealed that the average annual precipitation in the area amounted to approximately 9.3 mm. Notably, January, November, and December were identified as the months with the highest recorded rainfall levels, as per data sourced from climate-data.org. The combination of these factors suggests that while Al-Lith typically experiences low annual precipitation, the region is highly susceptible to flash floods during specific months which are January, November, and December. This primary flood risk occurs due to significantly higher precipitation levels during these months, where rainfall is significantly higher. The last historical floods happened in November 2018 and December 2022.

**Table 3:** Annual average precipitation of Al Laith.

Month	Jan	Feb	Mar	Apr	May	Jun	Jul	Aug	Sep	Oct	Nov	Dec	Year
Average precipitation mm	20	6	7	11	8	2	2	4	3	8	20	21	9.3

As a combined result of the above, this study harnessed the power of the DEM to conduct an in-depth analysis of the study area's drainage network, stream orders, and topographical features. ArcGIS software facilitated the creation of informative slope, aspect, and hill shade maps, shedding light on the terrain’s characteristics and emphasizing flood vulnerabilities in Al-Lith Valley. Furthermore, the examination of monthly precipitation data unveiled the region’s average annual rainfall patterns, highlighting specific months of heightened precipitation (Table 4). These integrated findings contribute to a comprehensive understanding of the study area's hydrological and topographic dynamics, which are crucial for flood risk assessment and mitigation efforts.

**Table 4:** Some morphometric analyses of the study area.

Parameter	Results
Area	2890 Km^2^
Perimeter	388 km
Stream length (Lu)	3438 km
Drainage density (Dd)	1.19
Stream frequency (Fs)	3.32
Drainage intensity (Di)	2.79
Infiltration number (If)	3.95

#### Morphometric parameters analysis

In the assessment of the study area’s morphometric characteristics, several key parameters were examined to gain valuable insights into its drainage network and hydrological behavior.

##### Drainage density (Dd)

Drainage density (Dd) serves as a fundamental metric, calculated as the total length of streams within a drainage basin divided by its area (A). In the present research region, a notably low drainage density of 1.19 km^−1^ is observed, indicative of a scarcity of streams relative to the area’s expanse. This characteristic can be primarily attributed to the presence of erosion-resistant, fractured, and rough rock formations that facilitate accelerated water flow within the wadi^26^.

##### Stream frequency (Fs)

Stream frequency (Fs) signifies the abundance of streams within a specific area, quantified as the number of streams per unit area. In the studied domain, the stream frequency is calculated to be 3.32 km^2^, revealing a relatively low stream density. This implies a scarcity of streams per square kilometer, a phenomenon influenced by factors such as modest relief, permeable subsurface materials, and a heightened capacity for infiltration. These conditions collectively contribute to the profusion of streams within the region^48,49^.

##### Bifurcation ratio (Rb)

The bifurcation ratio (Rb) provides insights into the branching pattern within a watershed’s stream network. It is computed as the ratio of the number of streams of a given order to the number of streams of the order directly above it. The mean bifurcation ratio (Mbr) in the study area is determined to be 1.96, signifying a notable degree of branching within the watershed’s stream network^49^.

##### Infiltration number (If)

The infiltration number (If) represents a comprehensive metric evaluating the infiltration capacity of a watershed, factoring in both drainage density and stream frequency. In the research region, the calculated infiltration number is 3.95, categorizing it as exhibiting low infiltration numbers and high runoff potential. This observation underscores the area’s propensity for high runoff rates due to its limited infiltration capacity^49^.

##### Flood risk assessment and site suitability

The interplay of drainage density, stream frequency, bifurcation ratio, and infiltration number impart significant insights into the watershed's characteristics and hydrological behavior. Notably, the low drainage density, low stream frequency, high bifurcation ratio, and low infiltration number in the study area collectively contribute to elevated flood risk and heightened potential for runoff. This assessment underscores the imperative necessity for the implementation of effective flood mitigation measures within the region.

Furthermore, a holistic approach was applied by^50^ involving the interrelationship of bifurcation ratio, drainage frequency, and drainage density to evaluate the basin’s hazard potential. Based on this analysis, the studied basin is identified as having a considerable likelihood of experiencing flash floods.

Briefly, the comprehensive analysis of morphometric parameters reveals critical insights into the study area’s hydrological behavior and flood risk. The observed characteristics necessitate diligent attention to flood risk mitigation strategies and effective management practices within the region.

### Geophysical data processing and interpretation

In the aftermath of an extensive field survey conducted within the study area, a meticulous and structured data processing sequence is enacted. This sequence encompasses several crucial steps geared toward enhancing data consistency and reliability.

#### Data quality assessment

The initial phase of data processing revolves around the generation of apparent resistance curves employing the field data. These curves serve the pivotal function of identifying and rectifying any irregularities, with particular emphasis on anomalies encountered during the onset of electrical and electromagnetic tests. Aberrant readings undergo rigorous scrutiny and, where necessary, are expunged from the dataset to elevate the overall precision and fidelity of the information.

#### Utilization of data processing software

Subsequently, specialized data processing software tools come into play, specifically the “Interpex 1DIV”^51^ and “ZondTEM1D”^52^ programs. These meticulously designed programs take on the responsibility of processing data originating from electrical probes. The dataset encompasses critical information, including electrical resistance, and, in applicable scenarios, resistance and inductive polarization. The primary probe data collected from the study site serves as the foundational data set for this comprehensive processing (Fig. 3b, c).

#### Development of a multi-layers model

The third phase in the data processing continuum is marked by efforts to streamline the representation of multi-layered data into a more coherent and manageable form. This procedure necessitates the amalgamation of groups of closely associated resistance values into unified composite resistance layers. The primary objective is to streamline the dataset’s complexity while preserving its intrinsic geoelectric attributes and characteristics.

#### Characterization of geoelectric layers

The ultimate stage of data processing culminates in the meticulous characterization of geoelectric layers. This encompasses the precise determination of electrical resistivity values, layer thicknesses, and the depths of the discrete geoelectric strata. These defined parameters offer a comprehensive understanding of the geological and geophysical attributes of the study area.

### Geophysical insights

The geophysical investigation, with a specific focus on the vicinity proximate to the groundwater dam and the Wadi Al-Leith water station within Wadi Al-Laith, has yielded valuable insights. The primary aim was to harness the dam’s influence on nearby wells, thus mitigating the necessity for extensive station-to-well extensions. Concurrently, the presence of a fractured layer and the heterogeneous topography of the solid base rocks were meticulously documented.

**Figure 6:**
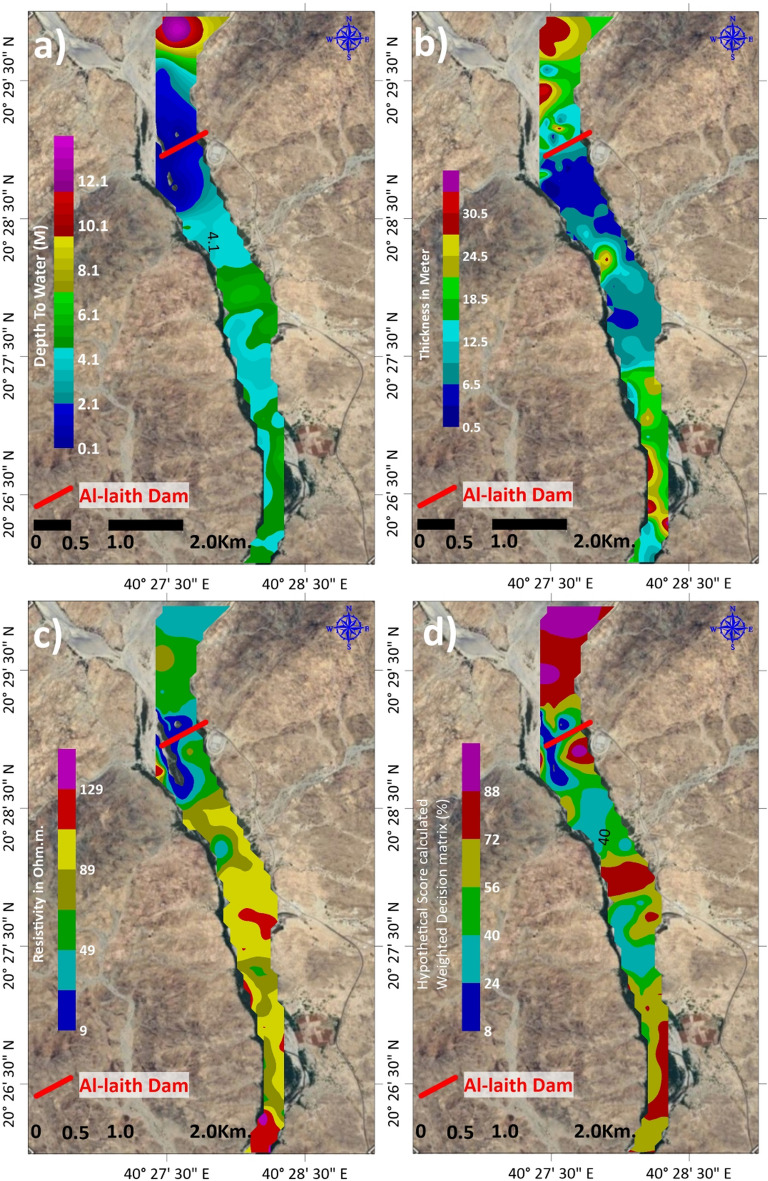
(**a**) Depth map to groundwater bearing layer, (**b**) thickness map of groundwater bearing layer, (**c**) resistivity distributions map of groundwater bearing layer, (**d**) map of hypothetical score calculation by geophysical weighted decision matrix [created using^26^.

The amalgamated findings underscore the existence of a substantial and adequately saturated sedimentary layer at select locations, coexisting alongside a cracked rocky layer harboring a discernible water content. It is pertinent to note that the predominant characteristic across the valley’s expanse is the prevalence of a notably thin sedimentary layer, characterized by limited water saturation (Fig. 6). It is clear from the interpretations that the depth of groundwater in the investigation area ranges from 0.5 to 14 m (Fig. 6a), and the thickness of the layer containing the water ranges between 0.3 and 33.63 m (Fig. 6b). The inference of the presence of groundwater was confirmed by an actual review of the results of the electrical resistance values, which ranged from 33.9 to 145 Ω.m (Fig. 6c).

In summation, the comprehensive geophysical investigation has unveiled the coexistence of well-saturated sedimentary layers and fractured rocky substrates across the study area. These findings constitute a pivotal resource for groundwater assessment and the judicious utilization of resources within the Wadi Al-Laith region.

### Matrix of comparative assessment of dam site suitability

#### Matrix of the effective geoelectrical model for dam site suitability

Matrices have been mentioned, as one of the means of evaluating the preference for identifying areas, in many studies that deal with environmental and water assessment processes for proposing or evaluating areas for constructing dams, such as^53–63^. The matrices differed in many of them depending on the parameters used and the data available (Tables S1 and S2 supplementary information documents). In this research, a somewhat unique matrix was designed based on the availability of data and the amount of correlation and complementarity between them.

In the comprehensive assessment of the geoelectric model of sublayers as an effective parameter in the suitability matrix for both the first and second regions, a series of parameters were carefully considered, each assigned a weight percentage to reflect its relative importance in the decision-making process. These parameters encompassed critical aspects of the geoelectrical model of sublayers and related factors, including layer resistivity (ρ), layer thickness (h), layer geometry, layer boundaries, electrode configuration, data quality and error estimation, inversion algorithm, geological constraints, and hydrogeological properties (Tables S3 and S4 supplementary information documents).

The weighted decision matrices for both regions were constructed by evaluating the effectiveness of each parameter for its effective power in site suitability. A hypothetical score calculation was then performed by multiplying the weight percentage by the effectiveness score for each parameter and summing these values for each region (Table 5, Fig. 6d). The results revealed that the second region excelled in suitability, achieving an impressive score of 60%, whereas the first region scored lower at 40%. This suggests that the second region is significantly more favorable for dam construction, as determined by the geoelectrical model of sublayers and its associated suitability parameters.

**Table 5:** Matrix of the effective geoelectrical model for dam site suitability.

Parameters	Weight %	Weight vector	Effective parameter of suitability matrix for building a dam for “the first region”	Effective parameter of suitability matrix for building a dam for “the second region”	Hypothetical score calculation weighted decision matrix for “the first region”	Hypothetical score calculation weighted decision matrix for “the second region”
Layer resistivity (ρ)	5	0.05	0.4	0.6	0.02	0.03
Layer thickness (h)	10	0.1	0.5	0.8	0.05	0.08
Layer geometry	10	0.1	0.5	0.8	0.05	0.08
Layer boundaries	5	0.05	0.5	0.9	0.025	0.045
Electrode configuration	1	0.01	0.2	0.6	0.002	0.006
Data quality and error estimation	5	0.05	0.5	0.5	0.025	0.025
Inversion algorithm	15	0.15	0.5	0.5	0.075	0.075
Geological constraints	20	0.2	0.4	0.9	0.08	0.18
Hydrogeological Properties (hydraulic conductivity, porosity, and groundwater flow properties)	15	0.15	0.5	0.5	0.075	0.075
Hypothetical score (geoelectrical model of sublayers for suitability parameter)					**40%**	**60%**

Significant values are given in bold.

#### Matrix of dam site suitability

In the evaluation of suitable locations for building a dam within the first and second regions, a set of parameters and their respective weightings were considered. These parameters included bifurcation ratio (Mbr), aspect, slope, hill shade of the study area, annual average precipitation, stream length (Lu), drainage density (Dd), stream frequency (Fs), drainage intensity (Di), infiltration number (If), flood possibilities, and the geoelectrical model of sublayers (Table 6). Each parameter was assigned a weight percentage reflecting its relative importance in the decision-making process. Subsequently, weighted decision matrices were created for both regions, where the quality of each parameter was assessed for each location.

**Table 6:** Matrix of comparative assessment of dam site suitability.

Parameters	Weight %	Weight vector	Quality for building a dam for “the first region”	Quality for building a dam for “the second region”	Hypothetical score calculation weighted decision matrix for “the first region”	Hypothetical score calculation weighted decision matrix for “the second region”
Bifurcation ratio (Mbr)	10	0.1	0.5	0.6	0.05	0.06
Aspect	5	0.05	0.5	0.6	0.025	0.03
Slope	10	0.1	0.4	0.7	0.04	0.07
Hill shade of the study area	5	0.05	0.6	0.7	0.03	0.035
Annual average precipitation	15	0.15	0.3	0.4	0.045	0.06
Stream length (Lu)	5	0.05	0.5	0.5	0.025	0.025
Drainage density (Dd)	10	0.1	0.4	0.5	0.04	0.05
Stream frequency (Fs)	10	0.1	0.4	0.5	0.04	0.05
Drainage intensity (Di)	5	0.05	0.3	0.4	0.015	0.02
Infiltration number (If)	10	0.1	0.6	0.7	0.06	0.07
Flood possibilities	10	0.1	0.5	0.6	0.05	0.06
Geoelectrical model of sublayers	5	0.05	0.4	0.6	0.02	0.03
Hypothetical score (suitability for building a dam)					**44%**	**56%**

Significant values are given in bold.

**Figure 7:**
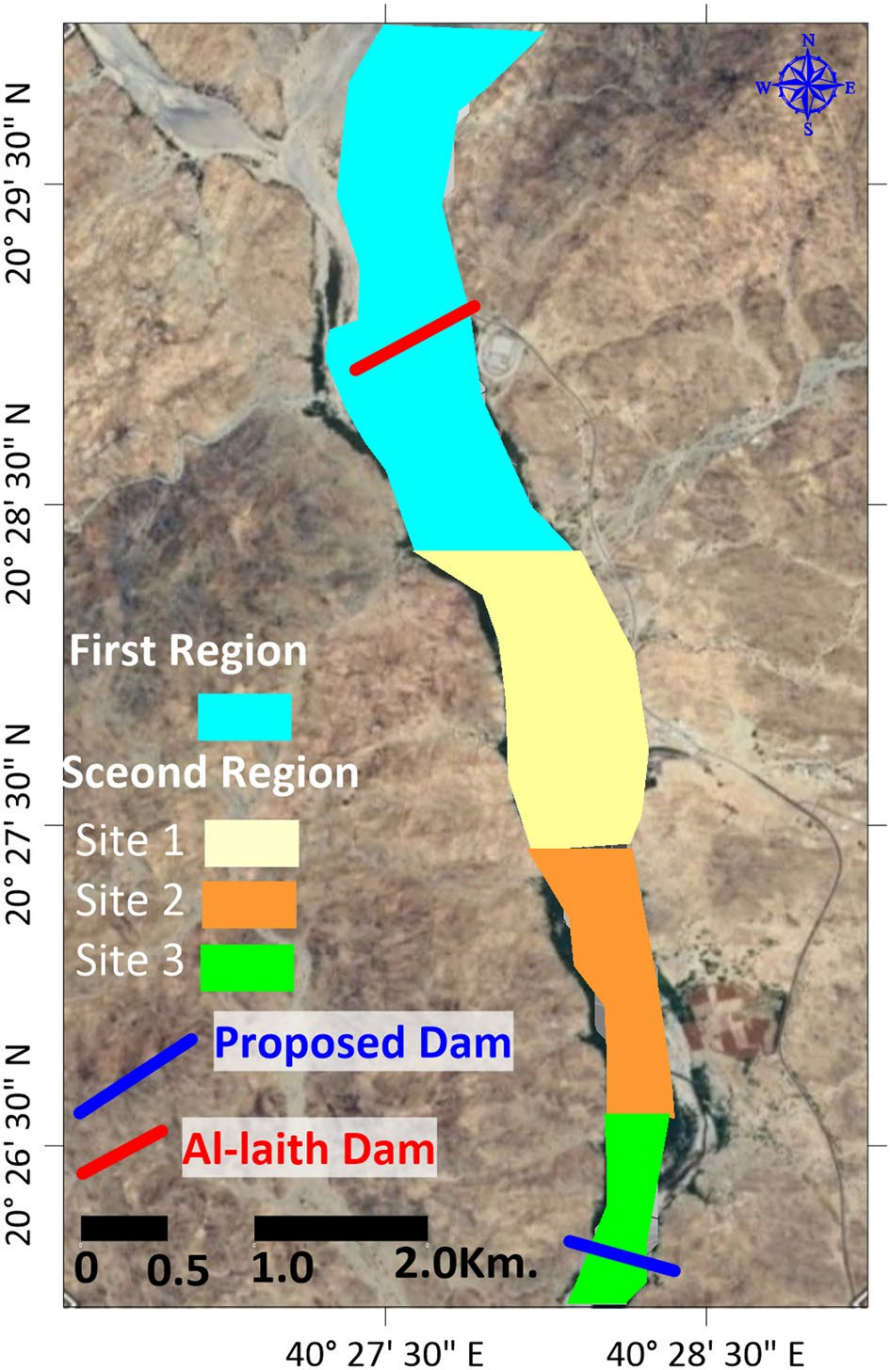
Map of hypothetical score calculation by hydrogeological and geophysical weighted decision matrix. Created using^26^.

The hypothetical score calculation was performed by multiplying the weight percentage by the quality score for each parameter and summing these values for each region. Based on this analysis, the first region, where the old dam was located, received a suitability score of 44%, while the second region scored higher at 56%, suggesting that the second region may be a more suitable option for building a dam according to the specified criteria (Fig. 7).

### Evaluating dam site suitability

The assessment conducted through matrix analysis has yielded valuable insights into the suitability of potential dam sites in the specified regions. These findings are rooted in a meticulous evaluation of various parameters and their weighted contributions to the overall suitability score. In this context, the first region emerged with a suitability score of 44%, while the second region demonstrated a notably higher score of 56%. This discrepancy in scores underscores a critical distinction between the two regions in terms of their potential for dam construction (Fig. 7).

The higher score awarded to the second region suggests that it may hold distinct advantages when measured against the specific criteria used for evaluation. These criteria, which include factors like bifurcation ratio (Mbr), aspect, slope, hill shade of the study area, annual average precipitation, stream length (Lu), drainage density (Dd), stream frequency (Fs), drainage intensity (Di), infiltration number (If), flood possibilities, and the geoelectrical model of sublayers depended on geoelectrical properties, geological constraints, and hydrogeological considerations, collectively indicate a higher level of suitability for dam construction in the second region. This implies that the second region offers a more promising and feasible prospect for establishing a dam infrastructure, aligning closely with the predefined objectives and prerequisites of the project. As such, the findings of this analysis provide a compelling rationale for considering the second region as the preferred choice for future dam construction endeavors.

### Parameters of proposed dam

Although it is impossible to completely eliminate the risk of flash floods, there are a variety of strategies to lessen it. For example, it is possible to identify the areas that are most vulnerable to the hazard by analyzing the drainage system, hydrologic modeling, and the local geology. Dams and canals are suggested solutions to the issue in addition to assisting in collecting and replenishing water for various reasons.

**Figure 8:**
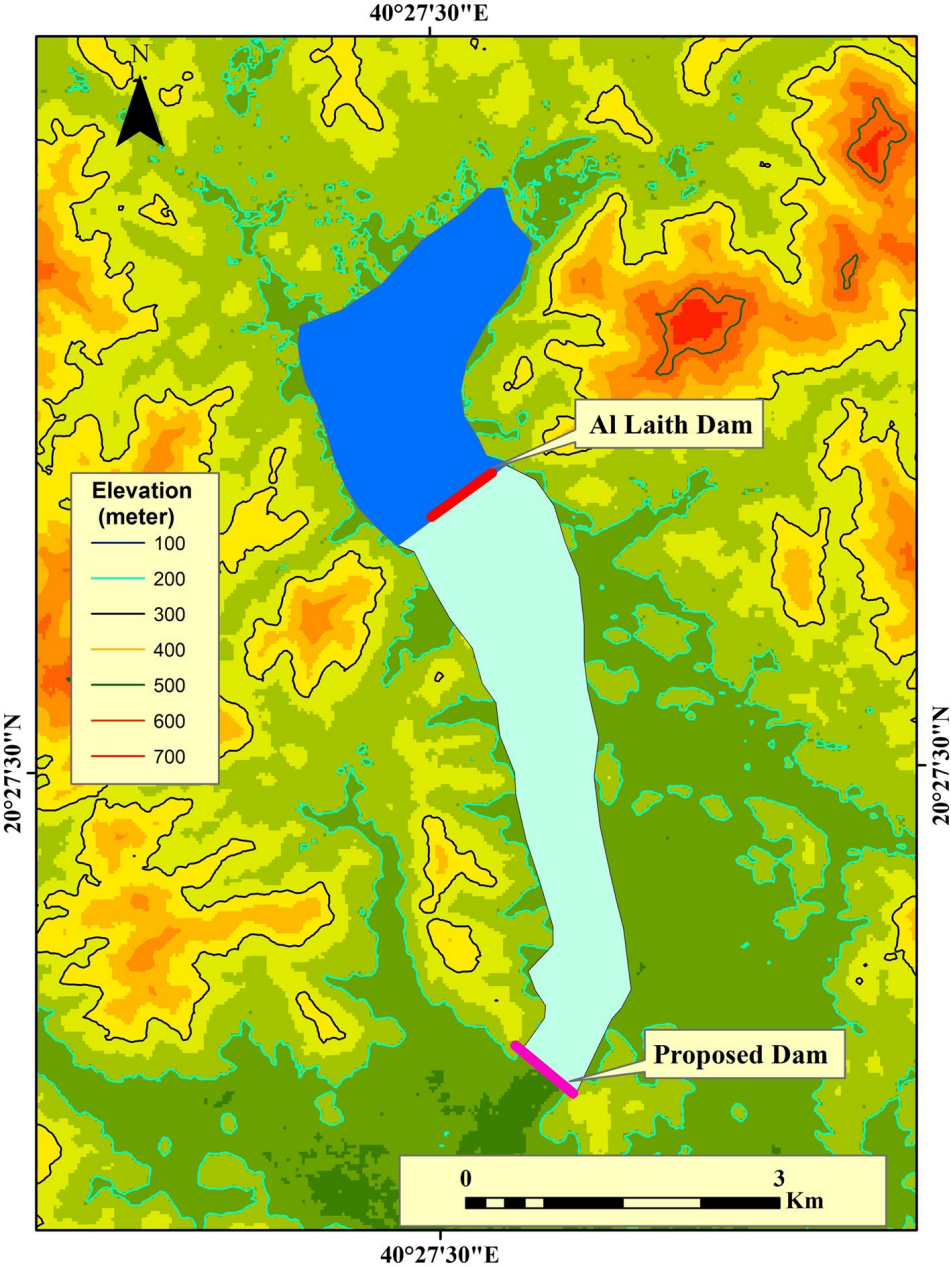
Map of the calculated storage-capacity volume of the proposed dam which is suggested for the area under investigation. Created using^34^.

The Al-Lith earthen dam in the study area collapsed on November 23, 2018, as a result of repeated rainstorm events in the upper part of Wadi Al-Lith in western Saudi Arabia^64^. An old Al-Lith dam was built as an altocumulus dam to solve this issue. Its height terminates at the earth's surface and its goal is to store groundwater to supply the wells dug above this dam. To supply a purification plant next to the old dam, a number of wells needed to be sunk at the top of the old dam (Fig. 8).

Based on the morphological analysis of the watershed and to reduce the risk of flash flooding^50^, the study of work suggests improving the proposed dam so that it can have a storage capacity of about 38,187,221.4 m^3^ and an area behind the dam of about 3,567,763.9 m^2^. Additionally, it may advocate building a supporting dam around 5 km south of the old Al-Lith Dam. Geologically, the site of the proposed and projected new Dam will be constructed on the two wadi sides with hard rock of quartz–diorite and no faults. The newly proposed dam will have a storage capacity of 114,624,651.1 m^3^, and its size will be 5,104,646.8 m^2^ (Fig. 8). According to GIS analysis, if the elevation map of the study area ranges from 122 to 617 m, the suggested proposed dam should measure between 230 and 280 m in height and 800 m in length.

## Discussion

The hydrogeological modeling conducted in this study leverages Digital Elevation Model (DEM) data to delineate the drainage network of Wadi Lith, revealing key insights into the region's susceptibility to flood hazards. The DEM analysis underscores the dominance of first-order streams (SU1) in the area, indicating a heightened vulnerability to drainage-related issues. The slope, aspect, and hill shade maps generated using ArcGIS further enhance our understanding of the region’s topography. The slope map highlights areas of steep inclinations, the aspect map shows a predominance of south-facing slopes, and the hill shade map vividly portrays the valley’s topographical features, emphasizing the Al-Lith Valley’s susceptibility to floods.

The analysis of morphometric parameters offers a comprehensive understanding of the drainage characteristics and flood risks within the study area. Drainage density (Dd) shows a value of 1.19 km^−1^, the low drainage density indicates a scarcity of streams, attributed to erosion-resistant rock formations that facilitate rapid water flow, contributing to flood risk. Stream Frequency (Fs) shows at 3.32 km^2^, the relatively low stream frequency suggests limited stream presence, influenced by modest relief and high infiltration capacity. The bifurcation ratio (Rb) shows a mean value of 1.96 reflecting significant branching within the stream network, crucial for understanding flood dynamics. Infiltration number (If) illustrates the low infiltration number of 3.95 highlights a high runoff potential, underlining the area’s vulnerability to flash floods. These parameters collectively indicate an elevated flood risk and necessitate effective mitigation strategies.

The geophysical investigation, focusing on the area around the groundwater dam and Wadi Al-Leith water station, reveals the coexistence of well-saturated sedimentary layers and fractured rocky substrates. This duality is crucial for groundwater assessment and highlights the potential for utilizing these resources effectively. The identified groundwater depths (0.5–14 m) and layer thicknesses (0.3–33.63 m) are significant for planning water extraction and management strategies.

The matrix analysis for dam site suitability compares two regions, considering various hydrological, geological, and geoelectrical parameters. The geoelectrical model illustrates that the second region scores higher (60%) compared to the first (40%), indicating better suitability for dam construction based on geoelectrical properties. Overall suitability containing factors like bifurcation ratio, aspect, slope, and precipitation illustrates that the second region again scores higher (56%) versus the first (44%). This comprehensive evaluation suggests that the second region is more favorable for dam construction due to its advantageous geoelectrical and topographical characteristics.

Considering the historical collapse of the Al-Lith Dam in November 2018 and December 2022, the study proposes improvements to the dam structure to enhance its storage capacity and flood mitigation capability. The proposed dam should have a storage capacity of approximately 114,624,651.1 m^3^, with a height of 230–280 m and a length of 800 m. This strategic enhancement aims to bolster the region's flood resilience and water management efficiency.

The integrated hydrogeological, geophysical, and morphometric analyses provide a holistic understanding of the flood risks and water management challenges in Wadi Al-Lith. The proposed mitigation strategies, including the construction of a new dam, are grounded in comprehensive geospatial and geophysical data, ensuring their effectiveness in enhancing the region’s flood resilience and water resource management. This study underscores the importance of leveraging advanced geospatial techniques and comprehensive data analysis for effective flood risk mitigation in arid regions.

## Conclusion

The study offers a comprehensive evaluation of flood risk mitigation strategies in Wadi Al-Laith, Kingdom of Saudi Arabia (KSA), emphasizing the critical need to address flood risks in arid regions due to their severe impact on communities, infrastructure, livelihoods, and the economy.

By using the hydrological analysis, the investigation of the morphometric parameters revealed low drainage density, low stream frequency, a high bifurcation ratio, and a low infiltration number, indicating elevated flood risk and high runoff potential in Wadi Al-Laith. These characteristics highlight the need for effective flood risk management to protect communities and infrastructure.

By using geophysical investigation, data processing used specialized software^51,52^ to process electrical and electromagnetic probe data, ensuring accuracy by correcting field data irregularities. The “multi-layer model” was developed by consolidating resistance values and providing detailed information on electrical resistivity, layer thicknesses, and depths of geoelectric strata. Findings include a well-saturated sedimentary layer and a cracked rocky layer with water content, though a thin, less saturated sedimentary layer is predominant. The study area was divided into two regions for dam construction, with the proposed new dam site scoring 56% in suitability, higher than the old dam sites at 44%.

The study indicates the encouragement and support of combining hydrogeological and geophysical data to offer a thorough understanding of factors contributing to flash floods, including topography, drainage characteristics, and subsurface properties.

Long-term implications of constructing dams have environmental Impacts like (1) dams significantly alter natural water flow, which can impact downstream ecosystems. By regulating water flow, dams can reduce the frequency and severity of floods, but they may also reduce sediment transport, affecting riverine habitats and delta formations. (2) The creation of a reservoir can lead to the submersion of land, affecting local flora and fauna. In arid regions like Wadi Al-Laith, this could disrupt unique desert ecosystems (3) Stagnant water in reservoirs can lead to reduced water quality, promoting the growth of algae and affecting aquatic life.

Also, the long-term implications of constructing dams have a morphological response like (1) the dam will trap sediments, leading to sediment accumulation in the reservoir. This can reduce the dam’s storage capacity over time and necessitate periodic dredging. (2) downstream of the dam, reduced sediment supply can lead to channel erosion, altering the geomorphology of the riverbed and potentially impacting infrastructure and habitats.

While acknowledging the potential long-term environmental implications, the decision to propose dam construction is based on a comprehensive assessment of the specific context of Wadi Al-Laith a recommended advice for building an 800 m-long auxiliary dam with a height of 230–280 m, utilizing quartz–diorite rock. Our analysis of morphometric parameters indicates a high flood risk due to low drainage density, low stream frequency, high bifurcation ratio, and low infiltration number. A strategically placed dam can significantly mitigate these risks. Additionally, the selected dam site in the second region, utilizing sturdy quartz–diorite rock without faults, provides a stable foundation for the proposed structure, ensuring its long-term stability and effectiveness. The proposed auxiliary dam, with a detailed design considering height, diameter, relief holes, surface inclinations, and well placements, aims to enhance flood resilience while addressing the specific hydrological and geological conditions of the area.

In addition to proposing dam construction, our study considered several non-structural and nature-based solutions to mitigate flood risk in Wadi Al-Laith, The study underscores the need for a holistic approach to enhance water resource management and support agriculture, and flood risk mitigation in arid regions like KSA, where infrequent but devastating floods can occur. A holistic approach to flood risk mitigation in arid regions like Wadi Al-Lith in the Kingdom of Saudi Arabia should combine structural and non-structural measures to address both immediate flood threats and long-term resilience, considering the unique hydrological and climatic conditions. Key strategies include (1) integrated watershed management, involving catchment area analysis, land use planning, and soil and water conservation; (2) structural measures, such as building dams, flood channels, and retention basins; (3) non-structural measures, including advanced flood forecasting, community engagement, and sustainable water management policies; (4) geophysical and hydrological monitoring through continuous data collection and geophysical surveys; (5) ecosystem-based approaches, such as restoring natural floodplains and promoting green infrastructure; and (6) adaptive management and research to allow flexibility in strategies and support ongoing research. By integrating these measures, advanced monitoring, and active community involvement, a holistic approach can significantly enhance flood resilience in arid regions like Wadi Al-Lith, addressing immediate risks and building long-term sustainability and adaptability to climate change.

The research contributes to flood risk management discourse in KSA by presenting innovative approaches to dam site selection using geophysical and geomorphological modeling. While our study acknowledges the potential long-term environmental implications of dam construction, it also highlights the necessity of such infrastructure in the specific context of Wadi Al-Laith to ensure effective flood risk mitigation. It offers valuable insights and recommendations to protect communities and infrastructure in arid regions prone to flash floods, promoting sustainable development. Findings can guide policymakers, researchers, and practitioners in KSA and similar arid regions globally.

### Supplementary Information

Supplementary Tables.

## Data Availability

All data generated or analyzed during this study are included in this mnuscript.
