# Supplementary material for: Global insights on flood risk mitigation in arid regions using geomorphological and geophysical modeling from a local case study
Source: Sci Rep. 2024 Aug 28;14:19975. doi: 10.1038/s41598-024-69541-x (PMC11358407; doi:10.1038/s41598-024-69541-x)
Supplement: Supplementary file 1 — Supplementary Tables. [file 41598_2024_69541_MOESM1_ESM.pdf]

Supplementary Tables

Table S1. The available data used as parameters in matrix for first region.

| Sounding No. | E | N | Resistivity (ρ) of Water Bearing Layer | Thickness (h) of Water Bearing Layer | Depth to Water Bearing Layer | Geometry % of Water Bearing Layer | Boundaries of Water Bearing Layer | Electrode Configuration | Data Quality and Error Estimation | Inversion Algorithm | | | | | | Geological Constraints | hydraulic conductivity (m/s) | porosity % | groundwater flow properties) | | | |
|---|---|---|---|---|---|---|---|---|---|---|---|---|---|---|---|---|---|---|---|---|---|---|
| | | | | | | | | | | Forward Model | Observed Data | Objective Function | Optimization | Iterative Process | Uncertainty Estimation | | | | Transmissivity (T) (m^2/s) | Storage Coefficient (S) | Specific Yield (Sy) % | Hydraulic Gradient (m) |
| 1 | 40.46033 | 20.47643 | 15 | 5.5 | 1 | 0.18 | 0.814286 | 0.30303 | 0.08849 | 0.100202 | 0.100202 | 0.100202 | 0.100202 | 0.100202 | 0.100202 | 0.40404 | 143.3198 | 0.552632 | 85.58573 | 0.467611 | 0.087586 | 0.291262 |
| 2 | 40.45974 | 20.4783 | 0 | 0 | 0 | 0 | 0.038 | 0.030303 | 0.15 | 0.01002 | 0.01002 | 0.01002 | 0.01002 | 0.01002 | 0.01002 | 0.20202 | 0 | 0.000737 | 0 | 0 | 0.002 | 1.700971 |
| 3 | 40.45918 | 20.47921 | 0 | 0 | 0 | 0 | 0.038 | 0.030303 | 0.15 | 0.01002 | 0.01002 | 0.01002 | 0.01002 | 0.01002 | 0.01002 | 0.20202 | 0 | 0.000737 | 0 | 0 | 0.002 | 1.631068 |
| 4 | 40.45868 | 20.47919 | 0 | 0 | 0 | 0 | 0.038 | 0.030303 | 0.15 | 0.01002 | 0.01002 | 0.01002 | 0.01002 | 0.01002 | 0.01002 | 0.20202 | 0 | 0.000737 | 0 | 0 | 0.002 | 1.776699 |
| 5 | 40.45893 | 20.47961 | 0 | 0 | 0 | 0 | 0.038 | 0.030303 | 0.15 | 0.01002 | 0.01002 | 0.01002 | 0.01002 | 0.01002 | 0.01002 | 0.20202 | 0 | 0.000737 | 0 | 0 | 0.002 | 0.990291 |
| 6 | 40.45849 | 20.47961 | 0 | 0 | 0 | 0 | 0.038 | 0.030303 | 0.15 | 0.01002 | 0.01002 | 0.01002 | 0.01002 | 0.01002 | 0.01002 | 0.20202 | 0 | 0.000737 | 0 | 0 | 0.002 | 1.980583 |
| 7 | 40.45781 | 20.4797 | 139.2 | 1 | 0.6 | 0.176 | 0.521143 | 0.193939 | 0.08849 | 0.06413 | 0.06413 | 0.06413 | 0.06413 | 0.06413 | 0.06413 | 0.258586 | 91.7247 | 0.353684 | 35.05592 | 0.299271 | 0.037056 | 2.702913 |
| 8 | 40.45888 | 20.47996 | 0 | 0 | 0 | 0 | 0.038 | 0.030303 | 0.147571 | 0.01002 | 0.01002 | 0.01002 | 0.01002 | 0.01002 | 0.01002 | 0.20202 | 0 | 0.000737 | 0 | 0 | 0.002 | 0.902913 |
| 9 | 40.45829 | 20.4801 | 0 | 0 | 0 | 0 | 0.038 | 0.030303 | 0.144534 | 0.01002 | 0.01002 | 0.01002 | 0.01002 | 0.01002 | 0.01002 | 0.20202 | 0 | 0.000737 | 0 | 0 | 0.002 | 0.640777 |
| 10 | 40.4576 | 20.48012 | 43.2 | 29.1 | 0.5 | 0.175 | 0.502143 | 0.186869 | 0.091093 | 0.061791 | 0.061791 | 0.061791 | 0.061791 | 0.061791 | 0.061791 | 0.249158 | 88.38057 | 0.340789 | 32.54635 | 0.28836 | 0.034546 | 0.838835 |
| 11 | 40.45865 | 20.48048 | 0 | 0 | 0 | 0 | 0.038 | 0.030303 | 0.12753 | 0.01002 | 0.01002 | 0.01002 | 0.01002 | 0.01002 | 0.01002 | 0.20202 | 0 | 0.000737 | 0 | 0 | 0.002 | 0.803883 |
| 12 | 40.45818 | 20.48047 | 0 | 0 | 0 | 0 | 0.038 | 0.030303 | 0.12753 | 0.01002 | 0.01002 | 0.01002 | 0.01002 | 0.01002 | 0.01002 | 0.20202 | 0 | 0.000737 | 0 | 0 | 0.002 | 0.594175 |
| 13 | 40.45743 | 20.48058 | 114 | 22.5 | 1.5 | 0.185 | 1.14 | 0.424242 | 0.080249 | 0.140283 | 0.140283 | 0.140283 | 0.140283 | 0.140283 | 0.140283 | 0.565657 | 200.6478 | 0.773684 | 167.748 | 0.654656 | 0.169748 | 2.213592 |
| 14 | 40.4591 | 20.48089 | 12 | 1.5 | 1 | 0.18 | 0.814286 | 0.30303 | 0.085541 | 0.100202 | 0.100202 | 0.100202 | 0.100202 | 0.100202 | 0.100202 | 0.40404 | 143.3198 | 0.552632 | 85.58573 | 0.467611 | 0.087586 | 0.23301 |
| 15 | 40.45948 | 20.48114 | 0 | 10 | 1 | 0.18 | 0.814286 | 0.30303 | 0.084066 | 0.100202 | 0.100202 | 0.100202 | 0.100202 | 0.100202 | 0.100202 | 0.40404 | 143.3198 | 0.552632 | 85.58573 | 0.467611 | 0.087586 | 0 |
| 16 | 40.45995 | 20.48128 | 42 | 0.5 | 1.5 | 0.185 | 1.14 | 0.424242 | 0.07808 | 0.140283 | 0.140283 | 0.140283 | 0.140283 | 0.140283 | 0.140283 | 0.565657 | 200.6478 | 0.773684 | 167.748 | 0.654656 | 0.169748 | 0.815534 |
| 17 | 40.46038 | 20.48155 | 70.8 | 1.4 | 1 | 0.18 | 0.814286 | 0.30303 | 0.082765 | 0.100202 | 0.100202 | 0.100202 | 0.100202 | 0.100202 | 0.100202 | 0.40404 | 143.3198 | 0.552632 | 85.58573 | 0.467611 | 0.087586 | 1.374757 |
| 18 | 40.46079 | 20.48179 | 69 | 4 | 2 | 0.19 | 1.340857 | 0.49899 | 0.075911 | 0.165 | 0.165 | 0.165 | 0.165 | 0.165 | 0.165 | 0.66532 | 236 | 0.91 | 232.0667 | 0.77 | 0.234067 | 1.339806 |
| 19 | 40.46123 | 20.48193 | 84 | 5 | 2 | 0.19 | 1.340857 | 0.49899 | 0.075911 | 0.165 | 0.165 | 0.165 | 0.165 | 0.165 | 0.165 | 0.66532 | 236 | 0.91 | 232.0667 | 0.77 | 0.234067 | 1.631068 |
| 20 | 40.46165 | 20.48212 | 58.5 | 3 | 2 | 0.19 | 1.340857 | 0.49899 | 0.074393 | 0.165 | 0.165 | 0.165 | 0.165 | 0.165 | 0.165 | 0.66532 | 236 | 0.91 | 232.0667 | 0.77 | 0.234067 | 1.135922 |
| 21 | 40.45813 | 20.48091 | 0 | 0 | 0 | 0 | 0.038 | 0.030303 | 0.12753 | 0.01002 | 0.01002 | 0.01002 | 0.01002 | 0.01002 | 0.01002 | 0.20202 | 0 | 0.000737 | 0 | 0 | 0.002 | 0.932039 |
| 22 | 40.45846 | 20.48096 | 0 | 0 | 0 | 0 | 0.038 | 0.030303 | 0.12753 | 0.01002 | 0.01002 | 0.01002 | 0.01002 | 0.01002 | 0.01002 | 0.20202 | 0 | 0.000737 | 0 | 0 | 0.002 | 0.361165 |
| 23 | 40.4573 | 20.48102 | 69 | 2 | 1.5 | 0.185 | 1.14 | 0.424242 | 0.07808 | 0.140283 | 0.140283 | 0.140283 | 0.140283 | 0.140283 | 0.140283 | 0.565657 | 200.6478 | 0.773684 | 167.748 | 0.654656 | 0.169748 | 1.339806 |

| | | | | | | | | | | | | | | | | | | | | | | |
|---|---|---|---|---|---|---|---|---|---|---|---|---|---|---|---|---|---|---|---|---|---|---|
| 24 | 40.4583 | 20.48132 | 0 | 0 | 0 | 0 | 0.038 | 0.030303 | 0.12753 | 0.01002 | 0.01002 | 0.01002 | 0.01002 | 0.01002 | 0.01002 | 0.20202 | 0 | 0.000737 | 0 | 0 | 0.002 | 3 |
| 25 | 40.45781 | 20.48132 | 0 | 0 | 0 | 0 | 0.038 | 0.030303 | 0.12753 | 0.01002 | 0.01002 | 0.01002 | 0.01002 | 0.01002 | 0.01002 | 0.20202 | 0 | 0.000737 | 0 | 0 | 0.002 | 0.495146 |
| 26 | 40.45703 | 20.48148 | 51 | 3.3 | 1.7 | 0.187 | 1.292 | 0.480808 | 0.07808 | 0.158988 | 0.158988 | 0.158988 | 0.158988 | 0.158988 | 0.158988 | 0.641077 | 227.4008 | 0.876842 | 215.463 | 0.741943 | 0.217463 | 0.990291 |
| 27 | 40.45815 | 20.48173 | 0 | 0 | 0 | 0 | 0.038 | 0.030303 | 0.118421 | 0.01002 | 0.01002 | 0.01002 | 0.01002 | 0.01002 | 0.01002 | 0.20202 | 0 | 0.000737 | 0 | 0 | 0.002 | 0.227184 |
| 28 | 40.45752 | 20.48178 | 0 | 0 | 0 | 0 | 0.038 | 0.030303 | 0.116599 | 0.01002 | 0.01002 | 0.01002 | 0.01002 | 0.01002 | 0.01002 | 0.20202 | 0 | 0.000737 | 0 | 0 | 0.002 | 0.262136 |
| 29 | 40.45701 | 20.48205 | 57 | 10.5 | 1 | 0.18 | 0.814286 | 0.30303 | 0.081984 | 0.100202 | 0.100202 | 0.100202 | 0.100202 | 0.100202 | 0.100202 | 0.40404 | 143.3198 | 0.552632 | 85.58573 | 0.467611 | 0.087586 | 1.106796 |
| 30 | 40.4578 | 20.48207 | 15 | 4.5 | 0.5 | 0.175 | 0.502143 | 0.186869 | 0.091093 | 0.061791 | 0.061791 | 0.061791 | 0.061791 | 0.061791 | 0.061791 | 0.249158 | 88.38057 | 0.340789 | 32.54635 | 0.28836 | 0.034546 | 0.291262 |
| 31 | 40.45823 | 20.4823 | 0 | 0 | 0 | 0 | 0.038 | 0.030303 | 0.116599 | 0.01002 | 0.01002 | 0.01002 | 0.01002 | 0.01002 | 0.01002 | 0.20202 | 0 | 0.000737 | 0 | 0 | 0.002 | 0.087379 |
| 32 | 40.45747 | 20.48236 | 0 | 0 | 0 | 0 | 0.038 | 0.030303 | 0.100202 | 0.01002 | 0.01002 | 0.01002 | 0.01002 | 0.01002 | 0.01002 | 0.20202 | 0 | 0.000737 | 0 | 0 | 0.002 | 0.23301 |
| 33 | 40.45812 | 20.4827 | 0 | 0 | 0 | 0 | 0.038 | 0.030303 | 0.097166 | 0.01002 | 0.01002 | 0.01002 | 0.01002 | 0.01002 | 0.01002 | 0.20202 | 0 | 0.000737 | 0 | 0 | 0.002 | 0.378641 |
| 34 | 40.45811 | 20.48322 | 0 | 0 | 0 | 0 | 0.038 | 0.030303 | 0.097166 | 0.01002 | 0.01002 | 0.01002 | 0.01002 | 0.01002 | 0.01002 | 0.20202 | 0 | 0.000737 | 0 | 0 | 0.002 | 0.699029 |
| 35 | 40.45723 | 20.48344 | 15 | 14.5 | 0.5 | 0.175 | 0.502143 | 0.186869 | 0.091093 | 0.061791 | 0.061791 | 0.061791 | 0.061791 | 0.061791 | 0.061791 | 0.249158 | 88.38057 | 0.340789 | 32.54635 | 0.28836 | 0.034546 | 0.291262 |
| 36 | 40.45786 | 20.48362 | 0 | 0 | 0 | 0 | 0.038 | 0.030303 | 0.097166 | 0.01002 | 0.01002 | 0.01002 | 0.01002 | 0.01002 | 0.01002 | 0.20202 | 0 | 0.000737 | 0 | 0 | 0.002 | 1.631068 |
| 37 | 40.45761 | 20.4838 | 0 | 0 | 0 | 0 | 0.038 | 0.030303 | 0.097166 | 0.01002 | 0.01002 | 0.01002 | 0.01002 | 0.01002 | 0.01002 | 0.20202 | 0 | 0.000737 | 0 | 0 | 0.002 | 0.757282 |
| 38 | 40.45773 | 20.48429 | 0 | 0 | 0 | 0 | 0.038 | 0.030303 | 0.093696 | 0.01002 | 0.01002 | 0.01002 | 0.01002 | 0.01002 | 0.01002 | 0.20202 | 0 | 0.000737 | 0 | 0 | 0.002 | 0.699029 |
| 39 | 40.45727 | 20.48433 | 0 | 0 | 0 | 0 | 0.038 | 0.030303 | 0.091093 | 0.01002 | 0.01002 | 0.01002 | 0.01002 | 0.01002 | 0.01002 | 0.20202 | 0 | 0.000737 | 0 | 0 | 0.002 | 0.466019 |
| 40 | 40.45893 | 20.48452 | 42 | 3 | 1 | 0.18 | 0.814286 | 0.30303 | 0.081203 | 0.100202 | 0.100202 | 0.100202 | 0.100202 | 0.100202 | 0.100202 | 0.40404 | 143.3198 | 0.552632 | 85.58573 | 0.467611 | 0.087586 | 0.815534 |
| 41 | 40.4573 | 20.48469 | 0 | 0 | 0 | 0 | 0.038 | 0.030303 | 0.091093 | 0.01002 | 0.01002 | 0.01002 | 0.01002 | 0.01002 | 0.01002 | 0.20202 | 0 | 0.000737 | 0 | 0 | 0.002 | 1.747573 |
| 42 | 40.45931 | 20.48489 | 0 | 0 | 0 | 0 | 0.038 | 0.030303 | 0.091093 | 0.01002 | 0.01002 | 0.01002 | 0.01002 | 0.01002 | 0.01002 | 0.20202 | 0 | 0.000737 | 0 | 0 | 0.002 | 0.932039 |
| 43 | 40.45886 | 20.48498 | 57 | 3 | 2 | 0.19 | 1.340857 | 0.49899 | 0.069838 | 0.165 | 0.165 | 0.165 | 0.165 | 0.165 | 0.165 | 0.66532 | 236 | 0.91 | 232.0667 | 0.77 | 0.234067 | 1.106796 |
| 44 | 40.45857 | 20.48515 | 26.4 | 4.2 | 1 | 0.18 | 0.814286 | 0.30303 | 0.081203 | 0.100202 | 0.100202 | 0.100202 | 0.100202 | 0.100202 | 0.100202 | 0.40404 | 143.3198 | 0.552632 | 85.58573 | 0.467611 | 0.087586 | 0.512621 |
| 45 | 40.45731 | 20.48515 | 0 | 0 | 0 | 0 | 0.038 | 0.030303 | 0.091093 | 0.01002 | 0.01002 | 0.01002 | 0.01002 | 0.01002 | 0.01002 | 0.20202 | 0 | 0.000737 | 0 | 0 | 0.002 | 0.466019 |
| 46 | 40.45803 | 20.48528 | 12 | 15 | 1 | 0.18 | 0.814286 | 0.30303 | 0.081116 | 0.100202 | 0.100202 | 0.100202 | 0.100202 | 0.100202 | 0.100202 | 0.40404 | 143.3198 | 0.552632 | 85.58573 | 0.467611 | 0.087586 | 0.23301 |
| 47 | 40.45929 | 20.4853 | 0 | 0 | 0 | 0 | 0.038 | 0.030303 | 0.091093 | 0.01002 | 0.01002 | 0.01002 | 0.01002 | 0.01002 | 0.01002 | 0.20202 | 0 | 0.000737 | 0 | 0 | 0.002 | 1.92233 |
| 48 | 40.4587 | 20.48548 | 21 | 24.5 | 1.5 | 0.185 | 1.14 | 0.424242 | 0.07808 | 0.140283 | 0.140283 | 0.140283 | 0.140283 | 0.140283 | 0.140283 | 0.565657 | 200.6478 | 0.773684 | 167.748 | 0.654656 | 0.169748 | 0.407767 |
| 49 | 40.45733 | 20.48559 | 0 | 0 | 0 | 0 | 0.038 | 0.030303 | 0.091093 | 0.01002 | 0.01002 | 0.01002 | 0.01002 | 0.01002 | 0.01002 | 0.20202 | 0 | 0.000737 | 0 | 0 | 0.002 | 0.349515 |
| 50 | 40.45955 | 20.48562 | 0 | 0 | 0 | 0 | 0.038 | 0.030303 | 0.091093 | 0.01002 | 0.01002 | 0.01002 | 0.01002 | 0.01002 | 0.01002 | 0.20202 | 0 | 0.000737 | 0 | 0 | 0.002 | 0.699029 |
| 51 | 40.457 | 20.4856 | 0 | 0 | 0 | 0 | 0.038 | 0.030303 | 0.091093 | 0.01002 | 0.01002 | 0.01002 | 0.01002 | 0.01002 | 0.01002 | 0.20202 | 0 | 0.000737 | 0 | 0 | 0.002 | 1.223301 |
| 52 | 40.45779 | 20.48573 | 32.4 | 13 | 1.2 | 0.182 | 1.042286 | 0.387879 | 0.080249 | 0.128259 | 0.128259 | 0.128259 | 0.128259 | 0.128259 | 0.128259 | 0.517172 | 183.4494 | 0.707368 | 140.2237 | 0.598543 | 0.142224 | 0.629126 |
| 53 | 40.45918 | 20.48574 | 0 | 0 | 0 | 0 | 0.038 | 0.030303 | 0.091093 | 0.01002 | 0.01002 | 0.01002 | 0.01002 | 0.01002 | 0.01002 | 0.20202 | 0 | 0.000737 | 0 | 0 | 0.002 | 0.466019 |
| 54 | 40.45823 | 20.48576 | 15 | 11.2 | 1.8 | 0.188 | 1.319143 | 0.490909 | 0.075911 | 0.162328 | 0.162328 | 0.162328 | 0.162328 | 0.162328 | 0.162328 | 0.654545 | 232.1781 | 0.895263 | 224.6112 | 0.75753 | 0.226611 | 0.291262 |
| 55 | 40.45879 | 20.48595 | 9 | 26.4 | 0.6 | 0.176 | 0.521143 | 0.193939 | 0.08849 | 0.06413 | 0.06413 | 0.06413 | 0.06413 | 0.06413 | 0.06413 | 0.258586 | 91.7247 | 0.353684 | 35.05592 | 0.299271 | 0.037056 | 0.174757 |
| 56 | 40.45909 | 20.48598 | 33 | 29.5 | 0.5 | 0.175 | 0.502143 | 0.186869 | 0.090659 | 0.061791 | 0.061791 | 0.061791 | 0.061791 | 0.061791 | 0.061791 | 0.249158 | 88.38057 | 0.340789 | 32.54635 | 0.28836 | 0.034546 | 0.640777 |
| 57 | 40.45782 | 20.486 | 33 | 12 | 1 | 0.18 | 0.814286 | 0.30303 | 0.081116 | 0.100202 | 0.100202 | 0.100202 | 0.100202 | 0.100202 | 0.100202 | 0.40404 | 143.3198 | 0.552632 | 85.58573 | 0.467611 | 0.087586 | 0.640777 |

| | | | | | | | | | | | | | | | | | | | | | |
|---|---|---|---|---|---|---|---|---|---|---|---|---|---|---|---|---|---|---|---|---|---|
| 58 | 40.45715 | 20.48602 | 0 | 0 | 0 | 0 | 0.038 | 0.030303 | 0.091093 | 0.01002 | 0.01002 | 0.01002 | 0.01002 | 0.01002 | 0.01002 | 0.20202 | 0 | 0.000737 | 0 | 0 | 0.002 | 0.407767 |
| 59 | 40.45941 | 20.48607 | 33 | 33.4 | 0.6 | 0.176 | 0.521143 | 0.193939 | 0.08849 | 0.06413 | 0.06413 | 0.06413 | 0.06413 | 0.06413 | 0.06413 | 0.258586 | 91.7247 | 0.353684 | 35.05592 | 0.299271 | 0.037056 | 0.640777 |
| 60 | 40.45819 | 20.48612 | 36 | 13 | 1 | 0.18 | 0.814286 | 0.30303 | 0.081116 | 0.100202 | 0.100202 | 0.100202 | 0.100202 | 0.100202 | 0.100202 | 0.40404 | 143.3198 | 0.552632 | 85.58573 | 0.467611 | 0.087586 | 0.699029 |
| 61 | 40.45848 | 20.48617 | 66 | 17 | 1 | 0.18 | 0.814286 | 0.30303 | 0.081116 | 0.100202 | 0.100202 | 0.100202 | 0.100202 | 0.100202 | 0.100202 | 0.40404 | 143.3198 | 0.552632 | 85.58573 | 0.467611 | 0.087586 | 1.281553 |
| 62 | 40.459 | 20.48625 | 69 | 14.5 | 0.5 | 0.175 | 0.502143 | 0.186869 | 0.090659 | 0.061791 | 0.061791 | 0.061791 | 0.061791 | 0.061791 | 0.061791 | 0.249158 | 88.38057 | 0.340789 | 32.54635 | 0.28836 | 0.034546 | 1.339806 |
| 63 | 40.45781 | 20.48626 | 78 | 4 | 1 | 0.18 | 0.814286 | 0.30303 | 0.081116 | 0.100202 | 0.100202 | 0.100202 | 0.100202 | 0.100202 | 0.100202 | 0.40404 | 143.3198 | 0.552632 | 85.58573 | 0.467611 | 0.087586 | 1.514563 |
| 64 | 40.45869 | 20.48634 | 36 | 34 | 1 | 0.18 | 0.868571 | 0.323232 | 0.080249 | 0.106883 | 0.106883 | 0.106883 | 0.106883 | 0.106883 | 0.106883 | 0.430976 | 152.8745 | 0.589474 | 97.37755 | 0.498785 | 0.099378 | 0.699029 |
| 65 | 40.45809 | 20.48643 | 69 | 22 | 1 | 0.18 | 0.868571 | 0.323232 | 0.080249 | 0.106883 | 0.106883 | 0.106883 | 0.106883 | 0.106883 | 0.106883 | 0.430976 | 152.8745 | 0.589474 | 97.37755 | 0.498785 | 0.099378 | 1.339806 |
| 66 | 40.45846 | 20.4865 | 31.5 | 20.5 | 1 | 0.18 | 0.868571 | 0.323232 | 0.080249 | 0.106883 | 0.106883 | 0.106883 | 0.106883 | 0.106883 | 0.106883 | 0.430976 | 152.8745 | 0.589474 | 97.37755 | 0.498785 | 0.099378 | 0.61165 |
| 67 | 40.45898 | 20.48652 | 75 | 14 | 1 | 0.18 | 0.868571 | 0.323232 | 0.080249 | 0.106883 | 0.106883 | 0.106883 | 0.106883 | 0.106883 | 0.106883 | 0.430976 | 152.8745 | 0.589474 | 97.37755 | 0.498785 | 0.099378 | 1.456311 |
| 68 | 40.45932 | 20.48651 | 54 | 2.1 | 0.9 | 0.179 | 0.732857 | 0.272727 | 0.08849 | 0.090182 | 0.090182 | 0.090182 | 0.090182 | 0.090182 | 0.090182 | 0.363636 | 128.9879 | 0.497368 | 69.32444 | 0.42085 | 0.071324 | 1.048544 |
| 69 | 40.45723 | 20.4866 | 0 | 0 | 0 | 0 | 0.038 | 0.030303 | 0.091093 | 0.01002 | 0.01002 | 0.01002 | 0.01002 | 0.01002 | 0.01002 | 0.20202 | 0 | 0.000737 | 0 | 0 | 0.002 | 0.757282 |
| 70 | 40.45791 | 20.48675 | 66 | 6.8 | 1.2 | 0.182 | 1.042286 | 0.387879 | 0.080249 | 0.128259 | 0.128259 | 0.128259 | 0.128259 | 0.128259 | 0.128259 | 0.517172 | 183.4494 | 0.707368 | 140.2237 | 0.598543 | 0.142224 | 1.281553 |
| 71 | 40.45732 | 20.48703 | 0 | 0 | 0 | 0 | 0.038 | 0.030303 | 0.091093 | 0.01002 | 0.01002 | 0.01002 | 0.01002 | 0.01002 | 0.01002 | 0.20202 | 0 | 0.000737 | 0 | 0 | 0.002 | 0.553398 |
| 72 | 40.45705 | 20.48738 | 63 | 23.5 | 1.5 | 0.185 | 1.14 | 0.424242 | 0.07808 | 0.140283 | 0.140283 | 0.140283 | 0.140283 | 0.140283 | 0.140283 | 0.565657 | 200.6478 | 0.773684 | 167.748 | 0.654656 | 0.169748 | 1.223301 |
| 73 | 40.45764 | 20.48921 | 45 | 26.7 | 1.3 | 0.183 | 1.058571 | 0.393939 | 0.080249 | 0.130263 | 0.130263 | 0.130263 | 0.130263 | 0.130263 | 0.130263 | 0.525253 | 186.3158 | 0.718421 | 144.6399 | 0.607895 | 0.14664 | 0.873786 |
| 74 | 40.45776 | 20.49098 | 66 | 32 | 2 | 0.19 | 1.340857 | 0.49899 | 0.069838 | 0.165 | 0.165 | 0.165 | 0.165 | 0.165 | 0.165 | 0.66532 | 236 | 0.91 | 232.0667 | 0.77 | 0.234067 | 1.281553 |
| 75 | 40.45796 | 20.49287 | 76.5 | 3 | 1.5 | 0.185 | 1.14 | 0.424242 | 0.07808 | 0.140283 | 0.140283 | 0.140283 | 0.140283 | 0.140283 | 0.140283 | 0.565657 | 200.6478 | 0.773684 | 167.748 | 0.654656 | 0.169748 | 1.485437 |
| Average of Region 1 | | | 26.66 | 7.021333 | 0.632 | 0.099253 | 0.515244 | 0.199071 | 0.095817 | 0.065826 | 0.065826 | 0.065826 | 0.065826 | 0.065826 | 0.065826 | 0.338694 | 87.65441 | 0.338324 | 63.80995 | 0.285991 | 0.06581 | 0.928 |

Table S2. The available data used as parameters in matrix for second region.

| Sounding No. | E | N | Resistivity (ρ) of Water Bearing Layer | Thickness (h) of Water Bearing Layer | Depth to Water Bearing Layer | Geometry % of Water Bearing Layer | Boundaries of Water Bearing Layer | Electrode Configuration | Data Quality and Error Estimation | Inversion Algorithm | | | | | | Geological Constraints | hydraulic conductivity (m/s) | porosity % | groundwater flow properties) | | | |
|---|---|---|---|---|---|---|---|---|---|---|---|---|---|---|---|---|---|---|---|---|---|---|
| | | | | | | | | | | Forward Model | Observed Data | Objective Function | Optimization | Iterative Process | Uncertainty Estimation | | | | Transmissivity (T) (m^2/s) | Storage Coefficient (S) | Specific Yield (Sy) % | Hydraulic Gradient (m) |
| 76 | 40.45823 | 20.49461 | 72 | 25 | 5 | 0.22 | 1.221429 | 0.757576 | 0.027762 | 0.083502 | 0.083502 | 0.083502 | 0.083502 | 0.083502 | 0.083502 | 0.757576 | 119.4332 | 0.460526 | 59.43454 | 0.389676 | 0.061435 | 1.398058 |
| 77 | 40.45885 | 20.49636 | 39 | 15 | 12 | 0.29 | 1.507592 | 0.935065 | 0.009109 | 0.103065 | 0.103065 | 0.103065 | 0.103065 | 0.103065 | 0.103065 | 0.935065 | 147.4147 | 0.568421 | 90.54621 | 0.480972 | 0.092546 | 0.757282 |
| 78 | 40.45984 | 20.49796 | 42 | 17 | 14 | 0.31 | 1.612286 | 1 | 0.009109 | 0.110223 | 0.110223 | 0.110223 | 0.110223 | 0.110223 | 0.110223 | 1 | 157.6518 | 0.607895 | 103.5587 | 0.514372 | 0.105559 | 0.815534 |
| 79 | 40.46075 | 20.49951 | 39 | 12 | 11 | 0.28 | 1.458735 | 0.904762 | 0.009109 | 0.099725 | 0.099725 | 0.099725 | 0.099725 | 0.099725 | 0.099725 | 0.904762 | 142.6374 | 0.55 | 84.77257 | 0.465385 | 0.086773 | 0.757282 |

| | | | | | | | | | | | | | | | | | | | | | | |
|---|---|---|---|---|---|---|---|---|---|---|---|---|---|---|---|---|---|---|---|---|---|---|
| 80 | 40.46178 | 20.50106 | 27 | 13 | 7 | 0.24 | 1.465714 | 0.909091 | 0.009109 | 0.100202 | 0.100202 | 0.100202 | 0.100202 | 0.100202 | 0.100202 | 0.909091 | 143.3198 | 0.552632 | 85.58573 | 0.467611 | 0.087586 | 0.524272 |
| 81 | 40.46262 | 20.5027 | 33 | 14 | 7 | 0.24 | 1.465714 | 0.909091 | 0.009109 | 0.100202 | 0.100202 | 0.100202 | 0.100202 | 0.100202 | 0.100202 | 0.909091 | 143.3198 | 0.552632 | 85.58573 | 0.467611 | 0.087586 | 0.640777 |
| 82 | 40.46365 | 20.50421 | 30 | 11 | 11 | 0.28 | 1.458735 | 0.904762 | 0.009109 | 0.099725 | 0.099725 | 0.099725 | 0.099725 | 0.099725 | 0.099725 | 0.904762 | 142.6374 | 0.55 | 84.77257 | 0.465385 | 0.086773 | 0.582524 |
| 83 | 40.46497 | 20.50576 | 21 | 12 | 7 | 0.24 | 1.465714 | 0.909091 | 0.009109 | 0.100202 | 0.100202 | 0.100202 | 0.100202 | 0.100202 | 0.100202 | 0.909091 | 143.3198 | 0.552632 | 85.58573 | 0.467611 | 0.087586 | 0.407767 |
| 84 | 40.46059 | 20.4743 | 82.5 | 4 | 5 | 0.22 | 1.221429 | 0.757576 | 0.027762 | 0.083502 | 0.083502 | 0.083502 | 0.083502 | 0.083502 | 0.083502 | 0.757576 | 119.4332 | 0.460526 | 59.43454 | 0.389676 | 0.061435 | 1.601942 |
| 85 | 40.46231 | 20.4751 | 91.5 | 2.3 | 4 | 0.21 | 0.446694 | 0.277056 | 0.059861 | 0.030538 | 0.030538 | 0.030538 | 0.030538 | 0.030538 | 0.030538 | 0.277056 | 43.67843 | 0.168421 | 7.949187 | 0.14251 | 0.009949 | 1.776699 |
| 86 | 40.46473 | 20.47575 | 79.5 | 3.5 | 4 | 0.21 | 0.446694 | 0.277056 | 0.05856 | 0.030538 | 0.030538 | 0.030538 | 0.030538 | 0.030538 | 0.030538 | 0.277056 | 43.67843 | 0.168421 | 7.949187 | 0.14251 | 0.009949 | 1.543689 |
| 87 | 40.46137 | 20.47257 | 99 | 1 | 4 | 0.21 | 0.446694 | 0.277056 | 0.05856 | 0.030538 | 0.030538 | 0.030538 | 0.030538 | 0.030538 | 0.030538 | 0.277056 | 43.67843 | 0.168421 | 7.949187 | 0.14251 | 0.009949 | 1.92233 |
| 88 | 40.46247 | 20.47307 | 63 | 12.5 | 4 | 0.21 | 0.446694 | 0.277056 | 0.05856 | 0.030538 | 0.030538 | 0.030538 | 0.030538 | 0.030538 | 0.030538 | 0.277056 | 43.67843 | 0.168421 | 7.949187 | 0.14251 | 0.009949 | 1.223301 |
| 89 | 40.4638 | 20.47336 | 99 | 0.5 | 4 | 0.21 | 0.446694 | 0.277056 | 0.05856 | 0.030538 | 0.030538 | 0.030538 | 0.030538 | 0.030538 | 0.030538 | 0.277056 | 43.67843 | 0.168421 | 7.949187 | 0.14251 | 0.009949 | 1.92233 |
| 90 | 40.46606 | 20.47358 | 101.1 | -0.5 | 4.6 | 0.216 | 0.963184 | 0.597403 | 0.028629 | 0.065847 | 0.065847 | 0.065847 | 0.065847 | 0.065847 | 0.065847 | 0.597403 | 94.18161 | 0.363158 | 36.95906 | 0.307287 | 0.038959 | 1.963107 |
| 91 | 40.4627 | 20.4704 | 102 | 1 | 4 | 0.21 | 0.446694 | 0.277056 | 0.05856 | 0.030538 | 0.030538 | 0.030538 | 0.030538 | 0.030538 | 0.030538 | 0.277056 | 43.67843 | 0.168421 | 7.949187 | 0.14251 | 0.009949 | 1.980583 |
| 92 | 40.46419 | 20.47018 | 44.4 | 19.5 | 4 | 0.21 | 0.460653 | 0.285714 | 0.0583 | 0.031492 | 0.031492 | 0.031492 | 0.031492 | 0.031492 | 0.031492 | 0.285714 | 45.04338 | 0.173684 | 8.453774 | 0.146964 | 0.010454 | 0.862136 |
| 93 | 40.46497 | 20.47025 | 19.2 | 27.6 | 4.5 | 0.215 | 0.942245 | 0.584416 | 0.030364 | 0.064416 | 0.064416 | 0.064416 | 0.064416 | 0.064416 | 0.064416 | 0.584416 | 92.13418 | 0.355263 | 35.36961 | 0.300607 | 0.03737 | 0.372816 |
| 94 | 40.46724 | 20.47018 | 102.9 | 0.7 | 4 | 0.21 | 0.460653 | 0.285714 | 0.0583 | 0.031492 | 0.031492 | 0.031492 | 0.031492 | 0.031492 | 0.031492 | 0.285714 | 45.04338 | 0.173684 | 8.453774 | 0.146964 | 0.010454 | 1.998058 |
| 95 | 40.4627 | 20.46837 | 104.1 | 0.3 | 4 | 0.21 | 0.460653 | 0.285714 | 0.0583 | 0.031492 | 0.031492 | 0.031492 | 0.031492 | 0.031492 | 0.031492 | 0.285714 | 45.04338 | 0.173684 | 8.453774 | 0.146964 | 0.010454 | 2.021359 |
| 96 | 40.46403 | 20.46772 | 39 | 21 | 5.5 | 0.225 | 1.305184 | 0.809524 | 0.009109 | 0.089228 | 0.089228 | 0.089228 | 0.089228 | 0.089228 | 0.089228 | 0.809524 | 127.6229 | 0.492105 | 67.86502 | 0.416397 | 0.069865 | 0.757282 |
| 97 | 40.46552 | 20.46743 | 105 | 1 | 5 | 0.22 | 1.221429 | 0.757576 | 0.027762 | 0.083502 | 0.083502 | 0.083502 | 0.083502 | 0.083502 | 0.083502 | 0.757576 | 119.4332 | 0.460526 | 59.43454 | 0.389676 | 0.061435 | 2.038835 |
| 98 | 40.46763 | 20.46844 | 79.5 | 8.5 | 5 | 0.22 | 1.256327 | 0.779221 | 0.027762 | 0.085888 | 0.085888 | 0.085888 | 0.085888 | 0.085888 | 0.085888 | 0.779221 | 122.8456 | 0.473684 | 62.87931 | 0.40081 | 0.064879 | 1.543689 |
| 99 | 40.46325 | 20.46641 | 108 | 1 | 5.5 | 0.225 | 1.305184 | 0.809524 | 0.009109 | 0.089228 | 0.089228 | 0.089228 | 0.089228 | 0.089228 | 0.089228 | 0.809524 | 127.6229 | 0.492105 | 67.86502 | 0.416397 | 0.069865 | 2.097087 |
| 100 | 40.46473 | 20.46518 | 107.7 | 1 | 5 | 0.22 | 1.256327 | 0.779221 | 0.027762 | 0.085888 | 0.085888 | 0.085888 | 0.085888 | 0.085888 | 0.085888 | 0.779221 | 122.8456 | 0.473684 | 62.87931 | 0.40081 | 0.064879 | 2.091262 |
| 101 | 40.46653 | 20.46511 | 97.5 | 2.5 | 6 | 0.23 | 1.423837 | 0.883117 | 0.009109 | 0.09734 | 0.09734 | 0.09734 | 0.09734 | 0.09734 | 0.09734 | 0.883117 | 139.225 | 0.536842 | 80.76499 | 0.454251 | 0.082765 | 1.893204 |
| 102 | 40.46864 | 20.46555 | 96 | 3.5 | 6 | 0.23 | 1.423837 | 0.883117 | 0.009109 | 0.09734 | 0.09734 | 0.09734 | 0.09734 | 0.09734 | 0.09734 | 0.883117 | 139.225 | 0.536842 | 80.76499 | 0.454251 | 0.082765 | 1.864078 |
| 103 | 40.46364 | 20.46366 | 102 | 1.5 | 6 | 0.23 | 1.423837 | 0.883117 | 0.009109 | 0.09734 | 0.09734 | 0.09734 | 0.09734 | 0.09734 | 0.09734 | 0.883117 | 139.225 | 0.536842 | 80.76499 | 0.454251 | 0.082765 | 1.980583 |
| 104 | 40.46684 | 20.46279 | 106.5 | 0.5 | 4 | 0.21 | 0.460653 | 0.285714 | 0.056174 | 0.031492 | 0.031492 | 0.031492 | 0.031492 | 0.031492 | 0.031492 | 0.285714 | 45.04338 | 0.173684 | 8.453774 | 0.146964 | 0.010454 | 2.067961 |
| 105 | 40.46942 | 20.46345 | 104.1 | 3.3 | 4 | 0.21 | 0.474612 | 0.294372 | 0.056174 | 0.032447 | 0.032447 | 0.032447 | 0.032447 | 0.032447 | 0.032447 | 0.294372 | 46.40833 | 0.178947 | 8.973887 | 0.151417 | 0.010974 | 2.021359 |
| 106 | 40.46395 | 20.46149 | 111 | 0.5 | 5.5 | 0.225 | 1.305184 | 0.809524 | 0.009109 | 0.089228 | 0.089228 | 0.089228 | 0.089228 | 0.089228 | 0.089228 | 0.809524 | 127.6229 | 0.492105 | 67.86502 | 0.416397 | 0.069865 | 2.15534 |
| 107 | 40.46591 | 20.46149 | 105 | 4 | 4 | 0.21 | 0.474612 | 0.294372 | 0.056174 | 0.032447 | 0.032447 | 0.032447 | 0.032447 | 0.032447 | 0.032447 | 0.294372 | 46.40833 | 0.178947 | 8.973887 | 0.151417 | 0.010974 | 2.038835 |
| 108 | 40.46778 | 20.46193 | 115.5 | 1 | 4 | 0.21 | 0.488571 | 0.30303 | 0.056174 | 0.033401 | 0.033401 | 0.033401 | 0.033401 | 0.033401 | 0.033401 | 0.30303 | 47.77328 | 0.184211 | 9.509526 | 0.15587 | 0.01151 | 2.242718 |
| 109 | 40.46989 | 20.46229 | 109.5 | 1.8 | 5.7 | 0.227 | 1.352645 | 0.838961 | 0.009109 | 0.092473 | 0.092473 | 0.092473 | 0.092473 | 0.092473 | 0.092473 | 0.838961 | 132.2637 | 0.51 | 72.8904 | 0.431538 | 0.07489 | 2.126214 |
| 110 | 40.46591 | 20.45946 | 102.6 | 2.8 | 4 | 0.21 | 0.488571 | 0.30303 | 0.056174 | 0.033401 | 0.033401 | 0.033401 | 0.033401 | 0.033401 | 0.033401 | 0.30303 | 47.77328 | 0.184211 | 9.509526 | 0.15587 | 0.01151 | 1.992233 |
| 111 | 40.46724 | 20.45939 | 88.5 | 6.5 | 4 | 0.21 | 0.488571 | 0.30303 | 0.052053 | 0.033401 | 0.033401 | 0.033401 | 0.033401 | 0.033401 | 0.033401 | 0.30303 | 47.77328 | 0.184211 | 9.509526 | 0.15587 | 0.01151 | 1.718447 |
| 112 | 40.46802 | 20.45975 | 91.5 | 9 | 5 | 0.22 | 1.256327 | 0.779221 | 0.009109 | 0.085888 | 0.085888 | 0.085888 | 0.085888 | 0.085888 | 0.085888 | 0.779221 | 122.8456 | 0.473684 | 62.87931 | 0.40081 | 0.064879 | 1.776699 |
| 113 | 40.47005 | 20.45983 | 112.5 | 3 | 4.5 | 0.215 | 0.942245 | 0.584416 | 0.030364 | 0.064416 | 0.064416 | 0.064416 | 0.064416 | 0.064416 | 0.064416 | 0.584416 | 92.13418 | 0.355263 | 35.36961 | 0.300607 | 0.03737 | 2.184466 |

| | | | | | | | | | | | | | | | | | | | | | | |
|---|---|---|---|---|---|---|---|---|---|---|---|---|---|---|---|---|---|---|---|---|---|---|
| 114 | 40.46606 | 20.45751 | 108 | 5 | 4 | 0.21 | 0.488571 | 0.30303 | 0.052053 | 0.033401 | 0.033401 | 0.033401 | 0.033401 | 0.033401 | 0.033401 | 0.30303 | 47.77328 | 0.184211 | 9.509526 | 0.15587 | 0.01151 | 2.097087 |
| 115 | 40.46786 | 20.45751 | 117 | 2.5 | 4 | 0.21 | 0.488571 | 0.30303 | 0.052053 | 0.033401 | 0.033401 | 0.033401 | 0.033401 | 0.033401 | 0.033401 | 0.30303 | 47.77328 | 0.184211 | 9.509526 | 0.15587 | 0.01151 | 2.271845 |
| 116 | 40.47036 | 20.45794 | 103.5 | 7.5 | 4 | 0.21 | 0.488571 | 0.30303 | 0.052053 | 0.033401 | 0.033401 | 0.033401 | 0.033401 | 0.033401 | 0.033401 | 0.30303 | 47.77328 | 0.184211 | 9.509526 | 0.15587 | 0.01151 | 2.009709 |
| 117 | 40.46458 | 20.45585 | 73.5 | 17.5 | 4 | 0.21 | 0.488571 | 0.30303 | 0.052053 | 0.033401 | 0.033401 | 0.033401 | 0.033401 | 0.033401 | 0.033401 | 0.30303 | 47.77328 | 0.184211 | 9.509526 | 0.15587 | 0.01151 | 1.427184 |
| 118 | 40.46606 | 20.45577 | 88.5 | 13 | 4.5 | 0.215 | 0.942245 | 0.584416 | 0.029497 | 0.064416 | 0.064416 | 0.064416 | 0.064416 | 0.064416 | 0.064416 | 0.584416 | 92.13418 | 0.355263 | 35.36961 | 0.300607 | 0.03737 | 1.718447 |
| 119 | 40.46724 | 20.45577 | 70.5 | 15.5 | 4 | 0.21 | 0.488571 | 0.30303 | 0.052053 | 0.033401 | 0.033401 | 0.033401 | 0.033401 | 0.033401 | 0.033401 | 0.30303 | 47.77328 | 0.184211 | 9.509526 | 0.15587 | 0.01151 | 1.368932 |
| 120 | 40.46849 | 20.45614 | 64.5 | 17.5 | 4 | 0.21 | 0.488571 | 0.30303 | 0.052053 | 0.033401 | 0.033401 | 0.033401 | 0.033401 | 0.033401 | 0.033401 | 0.30303 | 47.77328 | 0.184211 | 9.509526 | 0.15587 | 0.01151 | 1.252427 |
| 121 | 40.47075 | 20.45635 | 82.5 | 14.5 | 4 | 0.21 | 0.558367 | 0.34632 | 0.052053 | 0.038172 | 0.038172 | 0.038172 | 0.038172 | 0.038172 | 0.038172 | 0.34632 | 54.59803 | 0.210526 | 12.42061 | 0.178138 | 0.014421 | 1.601942 |
| 122 | 40.46575 | 20.4544 | 76.5 | 17.5 | 4 | 0.21 | 0.558367 | 0.34632 | 0.052053 | 0.038172 | 0.038172 | 0.038172 | 0.038172 | 0.038172 | 0.038172 | 0.34632 | 54.59803 | 0.210526 | 12.42061 | 0.178138 | 0.014421 | 1.485437 |
| 123 | 40.46708 | 20.45447 | 124.5 | 1.5 | 5 | 0.22 | 1.256327 | 0.779221 | 0.009109 | 0.085888 | 0.085888 | 0.085888 | 0.085888 | 0.085888 | 0.085888 | 0.779221 | 122.8456 | 0.473684 | 62.87931 | 0.40081 | 0.064879 | 2.417476 |
| 124 | 40.46903 | 20.45483 | 67.5 | 15.5 | 4 | 0.21 | 0.558367 | 0.34632 | 0.052053 | 0.038172 | 0.038172 | 0.038172 | 0.038172 | 0.038172 | 0.038172 | 0.34632 | 54.59803 | 0.210526 | 12.42061 | 0.178138 | 0.014421 | 1.31068 |
| 125 | 40.47091 | 20.45519 | 67.5 | 21.5 | 4 | 0.21 | 0.837551 | 0.519481 | 0.034702 | 0.057259 | 0.057259 | 0.057259 | 0.057259 | 0.057259 | 0.057259 | 0.519481 | 81.89705 | 0.315789 | 27.94636 | 0.267206 | 0.029946 | 1.31068 |
| 126 | 40.4663 | 20.45281 | 124.5 | 1.5 | 6 | 0.23 | 1.423837 | 0.883117 | 0.009109 | 0.09734 | 0.09734 | 0.09734 | 0.09734 | 0.09734 | 0.09734 | 0.883117 | 139.225 | 0.536842 | 80.76499 | 0.454251 | 0.082765 | 2.417476 |
| 127 | 40.46794 | 20.45266 | 125.4 | 2.7 | 5 | 0.22 | 1.256327 | 0.779221 | 0.009109 | 0.085888 | 0.085888 | 0.085888 | 0.085888 | 0.085888 | 0.085888 | 0.779221 | 122.8456 | 0.473684 | 62.87931 | 0.40081 | 0.064879 | 2.434951 |
| 128 | 40.46919 | 20.45266 | 91.5 | 15.5 | 4 | 0.21 | 0.837551 | 0.519481 | 0.034702 | 0.057259 | 0.057259 | 0.057259 | 0.057259 | 0.057259 | 0.057259 | 0.519481 | 81.89705 | 0.315789 | 27.94636 | 0.267206 | 0.029946 | 1.776699 |
| 129 | 40.47122 | 20.45252 | 94.5 | 13.5 | 5 | 0.22 | 1.256327 | 0.779221 | 0.009109 | 0.085888 | 0.085888 | 0.085888 | 0.085888 | 0.085888 | 0.085888 | 0.779221 | 122.8456 | 0.473684 | 62.87931 | 0.40081 | 0.064879 | 1.834951 |
| 130 | 40.46786 | 20.45107 | 138 | 0.5 | 4 | 0.21 | 0.837551 | 0.519481 | 0.034702 | 0.057259 | 0.057259 | 0.057259 | 0.057259 | 0.057259 | 0.057259 | 0.519481 | 81.89705 | 0.315789 | 27.94636 | 0.267206 | 0.029946 | 2.679612 |
| 131 | 40.46966 | 20.45056 | 64.5 | 23 | 5.5 | 0.225 | 1.305184 | 0.809524 | 0.009109 | 0.089228 | 0.089228 | 0.089228 | 0.089228 | 0.089228 | 0.089228 | 0.809524 | 127.6229 | 0.492105 | 67.86502 | 0.416397 | 0.069865 | 1.252427 |
| 132 | 40.47177 | 20.45056 | 100.5 | 13.5 | 4 | 0.21 | 0.837551 | 0.519481 | 0.030364 | 0.057259 | 0.057259 | 0.057259 | 0.057259 | 0.057259 | 0.057259 | 0.519481 | 81.89705 | 0.315789 | 27.94636 | 0.267206 | 0.029946 | 1.951456 |
| 133 | 40.46802 | 20.44897 | 76.5 | 20.5 | 4 | 0.21 | 0.837551 | 0.519481 | 0.030364 | 0.057259 | 0.057259 | 0.057259 | 0.057259 | 0.057259 | 0.057259 | 0.519481 | 81.89705 | 0.315789 | 27.94636 | 0.267206 | 0.029946 | 1.485437 |
| 134 | 40.46974 | 20.44875 | 117 | 8.5 | 4 | 0.21 | 0.837551 | 0.519481 | 0.030364 | 0.057259 | 0.057259 | 0.057259 | 0.057259 | 0.057259 | 0.057259 | 0.519481 | 81.89705 | 0.315789 | 27.94636 | 0.267206 | 0.029946 | 2.271845 |
| 135 | 40.47169 | 20.44875 | 104.4 | 11.5 | 5.2 | 0.222 | 1.30658 | 0.81039 | 0.009109 | 0.089323 | 0.089323 | 0.089323 | 0.089323 | 0.089323 | 0.089323 | 0.81039 | 127.7594 | 0.492632 | 68.01027 | 0.416842 | 0.07001 | 2.027184 |
| 136 | 40.47005 | 20.44672 | 70.5 | 24.5 | 4 | 0.21 | 0.837551 | 0.519481 | 0.030364 | 0.057259 | 0.057259 | 0.057259 | 0.057259 | 0.057259 | 0.057259 | 0.519481 | 81.89705 | 0.315789 | 27.94636 | 0.267206 | 0.029946 | 1.368932 |
| 137 | 40.47185 | 20.44607 | 112.5 | 9.7 | 5.8 | 0.228 | 1.376376 | 0.85368 | 0.009109 | 0.094095 | 0.094095 | 0.094095 | 0.094095 | 0.094095 | 0.094095 | 0.85368 | 134.5842 | 0.518947 | 75.47039 | 0.439109 | 0.07747 | 2.184466 |
| 138 | 40.47044 | 20.44499 | 73.5 | 24.9 | 4.6 | 0.216 | 1.123714 | 0.69697 | 0.028629 | 0.076822 | 0.076822 | 0.076822 | 0.076822 | 0.076822 | 0.076822 | 0.69697 | 109.8785 | 0.423684 | 50.30539 | 0.358502 | 0.052305 | 1.427184 |
| 139 | 40.46935 | 20.44332 | 52.5 | 28 | 4.5 | 0.215 | 0.942245 | 0.584416 | 0.029497 | 0.064416 | 0.064416 | 0.064416 | 0.064416 | 0.064416 | 0.064416 | 0.584416 | 92.13418 | 0.355263 | 35.36961 | 0.300607 | 0.03737 | 1.019417 |
| 140 | 40.46763 | 20.44245 | 118.5 | 10.5 | 5 | 0.22 | 1.291224 | 0.800866 | 0.009109 | 0.088274 | 0.088274 | 0.088274 | 0.088274 | 0.088274 | 0.088274 | 0.800866 | 126.258 | 0.486842 | 66.42113 | 0.411943 | 0.068421 | 2.300971 |
| 141 | 40.4695 | 20.44216 | 97.5 | 16.5 | 4 | 0.21 | 0.837551 | 0.519481 | 0.030364 | 0.057259 | 0.057259 | 0.057259 | 0.057259 | 0.057259 | 0.057259 | 0.519481 | 81.89705 | 0.315789 | 27.94636 | 0.267206 | 0.029946 | 1.893204 |
| 142 | 40.47005 | 20.43985 | 52.5 | 31.9 | 4.6 | 0.216 | 1.123714 | 0.69697 | 0.028629 | 0.076822 | 0.076822 | 0.076822 | 0.076822 | 0.076822 | 0.076822 | 0.69697 | 109.8785 | 0.423684 | 50.30539 | 0.358502 | 0.052305 | 1.019417 |
| 143 | 40.4724 | 20.44166 | 106.5 | 11.5 | 5 | 0.22 | 1.291224 | 0.800866 | 0.009109 | 0.088274 | 0.088274 | 0.088274 | 0.088274 | 0.088274 | 0.088274 | 0.800866 | 126.258 | 0.486842 | 66.42113 | 0.411943 | 0.068421 | 2.067961 |
| 144 | 40.47232 | 20.43941 | 88.5 | 15.5 | 5 | 0.22 | 1.291224 | 0.800866 | 0.009109 | 0.088274 | 0.088274 | 0.088274 | 0.088274 | 0.088274 | 0.088274 | 0.800866 | 126.258 | 0.486842 | 66.42113 | 0.411943 | 0.068421 | 1.718447 |
| 145 | 40.46872 | 20.43608 | 112.5 | 13 | 4.5 | 0.215 | 0.942245 | 0.584416 | 0.028629 | 0.064416 | 0.064416 | 0.064416 | 0.064416 | 0.064416 | 0.064416 | 0.584416 | 92.13418 | 0.355263 | 35.36961 | 0.300607 | 0.03737 | 2.184466 |
| 146 | 40.47013 | 20.43789 | 145.5 | 2.5 | 5 | 0.22 | 1.291224 | 0.800866 | 0.009109 | 0.088274 | 0.088274 | 0.088274 | 0.088274 | 0.088274 | 0.088274 | 0.800866 | 126.258 | 0.486842 | 66.42113 | 0.411943 | 0.068421 | 2.825243 |
| 147 | 40.47247 | 20.43811 | 55.5 | 32.5 | 5 | 0.22 | 1.291224 | 0.800866 | 0.009109 | 0.088274 | 0.088274 | 0.088274 | 0.088274 | 0.088274 | 0.088274 | 0.800866 | 126.258 | 0.486842 | 66.42113 | 0.411943 | 0.068421 | 1.07767 |

| | | | | | | | | | | | | | | | | | | | | | | |
|---|---|---|---|---|---|---|---|---|---|---|---|---|---|---|---|---|---|---|---|---|---|---|
| 148 | 40.46716 | 20.43239 | 91.5 | 20.5 | 5 | 0.22 | 1.291224 | 0.800866 | 0.009109 | 0.088274 | 0.088274 | 0.088274 | 0.088274 | 0.088274 | 0.088274 | 0.800866 | 126.258 | 0.486842 | 66.42113 | 0.411943 | 0.068421 | 1.776699 |
| 149 | 40.46598 | 20.43094 | 99 | 19 | 5 | 0.22 | 1.291224 | 0.800866 | 0.009109 | 0.088274 | 0.088274 | 0.088274 | 0.088274 | 0.088274 | 0.088274 | 0.800866 | 126.258 | 0.486842 | 66.42113 | 0.411943 | 0.068421 | 1.92233 |
| 150 | 40.46716 | 20.43008 | 115.5 | 12.5 | 5 | 0.22 | 1.291224 | 0.800866 | 0.009109 | 0.088274 | 0.088274 | 0.088274 | 0.088274 | 0.088274 | 0.088274 | 0.800866 | 126.258 | 0.486842 | 66.42113 | 0.411943 | 0.068421 | 2.242718 |
| 151 | 40.47099 | 20.43369 | 133.5 | 2.1 | 4.9 | 0.219 | 1.197 | 0.742424 | 0.027762 | 0.081832 | 0.081832 | 0.081832 | 0.081832 | 0.081832 | 0.081832 | 0.742424 | 117.0445 | 0.451316 | 57.08093 | 0.381883 | 0.059081 | 2.592233 |
| 152 | 40.4724 | 20.43623 | 106.5 | 17.5 | 4 | 0.21 | 0.837551 | 0.519481 | 0.030364 | 0.057259 | 0.057259 | 0.057259 | 0.057259 | 0.057259 | 0.057259 | 0.519481 | 81.89705 | 0.315789 | 27.94636 | 0.267206 | 0.029946 | 2.067961 |
| 153 | 40.46395 | 20.42971 | 136.5 | 5.3 | 5.2 | 0.222 | 1.30658 | 0.81039 | 0.009109 | 0.089323 | 0.089323 | 0.089323 | 0.089323 | 0.089323 | 0.089323 | 0.81039 | 127.7594 | 0.492632 | 68.01027 | 0.416842 | 0.07001 | 2.650485 |
| 154 | 40.46348 | 20.42805 | 84 | 23 | 4 | 0.21 | 0.837551 | 0.519481 | 0.030364 | 0.057259 | 0.057259 | 0.057259 | 0.057259 | 0.057259 | 0.057259 | 0.519481 | 81.89705 | 0.315789 | 27.94636 | 0.267206 | 0.029946 | 1.631068 |
| 155 | 40.46489 | 20.4274 | 91.5 | 22 | 5.5 | 0.225 | 1.305184 | 0.809524 | 0.009109 | 0.089228 | 0.089228 | 0.089228 | 0.089228 | 0.089228 | 0.089228 | 0.809524 | 127.6229 | 0.492105 | 67.86502 | 0.416397 | 0.069865 | 1.776699 |
| 156 | 40.46638 | 20.42703 | 84 | 25.2 | 5.3 | 0.223 | 1.331706 | 0.825974 | 0.009109 | 0.091041 | 0.091041 | 0.091041 | 0.091041 | 0.091041 | 0.091041 | 0.825974 | 130.2163 | 0.502105 | 70.6512 | 0.424858 | 0.072651 | 1.631068 |
| 157 | 40.4627 | 20.4308 | 43.5 | 30.5 | 6 | 0.23 | 1.423837 | 0.883117 | 0.009109 | 0.09734 | 0.09734 | 0.09734 | 0.09734 | 0.09734 | 0.09734 | 0.883117 | 139.225 | 0.536842 | 80.76499 | 0.454251 | 0.082765 | 0.84466 |
| Average of Region 2 | | | 88.10854 | 11.24512 | 5.071951 | 0.22072 | 0.991757 | 0.615125 | 0.028733 | 0.067801 | 0.067801 | 0.067801 | 0.067801 | 0.067801 | 0.067801 | 0.615125 | 96.9756 | 0.373931 | 44.86349 | 0.316403 | 0.046863 | 1.710845 |

Table S3. The parameters used as a critical aspect for making the geoelectrical model of sublayers depending on its related factors (First region).

| Sounding No. | E | N | Weight Vector | | | | | | | | | | | Hypothetical Score (Geolectrical Model of Sublayers for Suitability parameter) |
|---|---|---|---|---|---|---|---|---|---|---|---|---|---|---|
| | | | Resistivity (ρ) of Water Bearing Layer | Thickness (h) of Water Bearing Layer | Geometry of Water Bearing Layer | Boundaries of Water Bearing Layer | Electrode Configuration | Data Quality and Error Estimation | Inversion Algorithm | Geological Constraints | hydraulic conductivity | porosity | groundwater flow properties) | |
| 1 | 40.46033 | 20.47643 | 0.03 | 0.075 | 0.075 | 0.0375 | 0.003 | 0.0375 | 0.1125 | 0.12 | 0.0375 | 0.0375 | 0.0375 | 0.603 |
| 2 | 40.45974 | 20.4783 | 0 | 0 | 0.0005 | 0.00175 | 0.0003 | 0.00375 | 0.01125 | 0.06 | 0 | 0.00005 | 0 | 0.0776 |
| 3 | 40.45918 | 20.47921 | 0 | 0 | 0.0005 | 0.00175 | 0.0003 | 0.00375 | 0.01125 | 0.06 | 0 | 0.00005 | 0 | 0.0776 |
| 4 | 40.45868 | 20.47919 | 0 | 0 | 0.0005 | 0.00175 | 0.0003 | 0.00375 | 0.01125 | 0.06 | 0 | 0.00005 | 0 | 0.0776 |
| 5 | 40.45893 | 20.47961 | 0 | 0 | 0.0005 | 0.00175 | 0.0003 | 0.00375 | 0.01125 | 0.06 | 0 | 0.00005 | 0 | 0.0776 |
| 6 | 40.45849 | 20.47961 | 0 | 0 | 0.0005 | 0.00175 | 0.0003 | 0.00375 | 0.01125 | 0.06 | 0 | 0.00005 | 0 | 0.0776 |
| 7 | 40.45781 | 20.4797 | 0.0192 | 0.048 | 0.048 | 0.024 | 0.00192 | 0.024 | 0.072 | 0.0768 | 0.024 | 0.024 | 0.024 | 0.38592 |
| 8 | 40.45888 | 20.47996 | 0 | 0 | 0.0005 | 0.00175 | 0.0003 | 0.00375 | 0.01125 | 0.06 | 0 | 0.00005 | 0 | 0.0776 |
| 9 | 40.45829 | 20.4801 | 0 | 0 | 0.0005 | 0.00175 | 0.0003 | 0.00375 | 0.01125 | 0.06 | 0 | 0.00005 | 0 | 0.0776 |
| 10 | 40.4576 | 20.48012 | 0.0185 | 0.04625 | 0.04625 | 0.023125 | 0.00185 | 0.023125 | 0.069375 | 0.074 | 0.023125 | 0.023125 | 0.023125 | 0.37185 |

| | | | | | | | | | | | | | | |
|---|---|---|---|---|---|---|---|---|---|---|---|---|---|---|
| 11 | 40.45865 | 20.48048 | 0 | 0 | 0.0005 | 0.00175 | 0.0003 | 0.00375 | 0.01125 | 0.06 | 0 | 0.00005 | 0 | 0.0776 |
| 12 | 40.45818 | 20.48047 | 0 | 0 | 0.0005 | 0.00175 | 0.0003 | 0.00375 | 0.01125 | 0.06 | 0 | 0.00005 | 0 | 0.0776 |
| 13 | 40.45743 | 20.48058 | 0.042 | 0.105 | 0.105 | 0.0525 | 0.0042 | 0.0525 | 0.1575 | 0.168 | 0.0525 | 0.0525 | 0.0525 | 0.8442 |
| 14 | 40.4591 | 20.48089 | 0.03 | 0.075 | 0.075 | 0.0375 | 0.003 | 0.0375 | 0.1125 | 0.12 | 0.0375 | 0.0375 | 0.0375 | 0.603 |
| 15 | 40.45948 | 20.48114 | 0.03 | 0.075 | 0.075 | 0.0375 | 0.003 | 0.0375 | 0.1125 | 0.12 | 0.0375 | 0.0375 | 0.0375 | 0.603 |
| 16 | 40.45995 | 20.48128 | 0.042 | 0.105 | 0.105 | 0.0525 | 0.0042 | 0.0525 | 0.1575 | 0.168 | 0.0525 | 0.0525 | 0.0525 | 0.8442 |
| 17 | 40.46038 | 20.48155 | 0.03 | 0.075 | 0.075 | 0.0375 | 0.003 | 0.0375 | 0.1125 | 0.12 | 0.0375 | 0.0375 | 0.0375 | 0.603 |
| 18 | 40.46079 | 20.48179 | 0.0494 | 0.1235 | 0.1235 | 0.06175 | 0.00494 | 0.06175 | 0.18525 | 0.1976 | 0.06175 | 0.06175 | 0.06175 | 0.99294 |
| 19 | 40.46123 | 20.48193 | 0.0494 | 0.1235 | 0.1235 | 0.06175 | 0.00494 | 0.06175 | 0.18525 | 0.1976 | 0.06175 | 0.06175 | 0.06175 | 0.99294 |
| 20 | 40.46165 | 20.48212 | 0.0494 | 0.1235 | 0.1235 | 0.06175 | 0.00494 | 0.06175 | 0.18525 | 0.1976 | 0.06175 | 0.06175 | 0.06175 | 0.99294 |
| 21 | 40.45813 | 20.48091 | 0 | 0 | 0.0005 | 0.00175 | 0.0003 | 0.00375 | 0.01125 | 0.06 | 0 | 0.00005 | 0 | 0.0776 |
| 22 | 40.45846 | 20.48096 | 0 | 0 | 0.0005 | 0.00175 | 0.0003 | 0.00375 | 0.01125 | 0.06 | 0 | 0.00005 | 0 | 0.0776 |
| 23 | 40.4573 | 20.48102 | 0.042 | 0.105 | 0.105 | 0.0525 | 0.0042 | 0.0525 | 0.1575 | 0.168 | 0.0525 | 0.0525 | 0.0525 | 0.8442 |
| 24 | 40.4583 | 20.48132 | 0 | 0 | 0.0005 | 0.00175 | 0.0003 | 0.00375 | 0.01125 | 0.06 | 0 | 0.00005 | 0 | 0.0776 |
| 25 | 40.45781 | 20.48132 | 0 | 0 | 0.0005 | 0.00175 | 0.0003 | 0.00375 | 0.01125 | 0.06 | 0 | 0.00005 | 0 | 0.0776 |
| 26 | 40.45703 | 20.48148 | 0.0476 | 0.119 | 0.119 | 0.0595 | 0.00476 | 0.0595 | 0.1785 | 0.1904 | 0.0595 | 0.0595 | 0.0595 | 0.95676 |
| 27 | 40.45815 | 20.48173 | 0 | 0 | 0.0005 | 0.00175 | 0.0003 | 0.00375 | 0.01125 | 0.06 | 0 | 0.00005 | 0 | 0.0776 |
| 28 | 40.45752 | 20.48178 | 0 | 0 | 0.0005 | 0.00175 | 0.0003 | 0.00375 | 0.01125 | 0.06 | 0 | 0.00005 | 0 | 0.0776 |
| 29 | 40.45701 | 20.48205 | 0.03 | 0.075 | 0.075 | 0.0375 | 0.003 | 0.0375 | 0.1125 | 0.12 | 0.0375 | 0.0375 | 0.0375 | 0.603 |
| 30 | 40.4578 | 20.48207 | 0.0185 | 0.04625 | 0.04625 | 0.023125 | 0.00185 | 0.023125 | 0.069375 | 0.074 | 0.023125 | 0.023125 | 0.023125 | 0.37185 |
| 31 | 40.45823 | 20.4823 | 0 | 0 | 0.0005 | 0.00175 | 0.0003 | 0.00375 | 0.01125 | 0.06 | 0 | 0.00005 | 0 | 0.0776 |
| 32 | 40.45747 | 20.48236 | 0 | 0 | 0.0005 | 0.00175 | 0.0003 | 0.00375 | 0.01125 | 0.06 | 0 | 0.00005 | 0 | 0.0776 |
| 33 | 40.45812 | 20.4827 | 0 | 0 | 0.0005 | 0.00175 | 0.0003 | 0.00375 | 0.01125 | 0.06 | 0 | 0.00005 | 0 | 0.0776 |
| 34 | 40.45811 | 20.48322 | 0 | 0 | 0.0005 | 0.00175 | 0.0003 | 0.00375 | 0.01125 | 0.06 | 0 | 0.00005 | 0 | 0.0776 |
| 35 | 40.45723 | 20.48344 | 0.0185 | 0.04625 | 0.04625 | 0.023125 | 0.00185 | 0.023125 | 0.069375 | 0.074 | 0.023125 | 0.023125 | 0.023125 | 0.37185 |
| 36 | 40.45786 | 20.48362 | 0 | 0 | 0.0005 | 0.00175 | 0.0003 | 0.00375 | 0.01125 | 0.06 | 0 | 0.00005 | 0 | 0.0776 |
| 37 | 40.45761 | 20.4838 | 0 | 0 | 0.0005 | 0.00175 | 0.0003 | 0.00375 | 0.01125 | 0.06 | 0 | 0.00005 | 0 | 0.0776 |
| 38 | 40.45773 | 20.48429 | 0 | 0 | 0.0005 | 0.00175 | 0.0003 | 0.00375 | 0.01125 | 0.06 | 0 | 0.00005 | 0 | 0.0776 |
| 39 | 40.45727 | 20.48433 | 0 | 0 | 0.0005 | 0.00175 | 0.0003 | 0.00375 | 0.01125 | 0.06 | 0 | 0.00005 | 0 | 0.0776 |
| 40 | 40.45893 | 20.48452 | 0.03 | 0.075 | 0.075 | 0.0375 | 0.003 | 0.0375 | 0.1125 | 0.12 | 0.0375 | 0.0375 | 0.0375 | 0.603 |
| 41 | 40.4573 | 20.48469 | 0 | 0 | 0.0005 | 0.00175 | 0.0003 | 0.00375 | 0.01125 | 0.06 | 0 | 0.00005 | 0 | 0.0776 |
| 42 | 40.45931 | 20.48489 | 0 | 0 | 0.0005 | 0.00175 | 0.0003 | 0.00375 | 0.01125 | 0.06 | 0 | 0.00005 | 0 | 0.0776 |
| 43 | 40.45886 | 20.48498 | 0.0494 | 0.1235 | 0.1235 | 0.06175 | 0.00494 | 0.06175 | 0.18525 | 0.1976 | 0.06175 | 0.06175 | 0.06175 | 0.99294 |
| 44 | 40.45857 | 20.48515 | 0.03 | 0.075 | 0.075 | 0.0375 | 0.003 | 0.0375 | 0.1125 | 0.12 | 0.0375 | 0.0375 | 0.0375 | 0.603 |

| | | | | | | | | | | | | | | |
|---|---|---|---|---|---|---|---|---|---|---|---|---|---|---|
| 45 | 40.45731 | 20.48515 | 0 | 0 | 0.0005 | 0.00175 | 0.0003 | 0.00375 | 0.01125 | 0.06 | 0 | 0.00005 | 0 | 0.0776 |
| 46 | 40.45803 | 20.48528 | 0.03 | 0.075 | 0.075 | 0.0375 | 0.003 | 0.0375 | 0.1125 | 0.12 | 0.0375 | 0.0375 | 0.0375 | 0.603 |
| 47 | 40.45929 | 20.4853 | 0 | 0 | 0.0005 | 0.00175 | 0.0003 | 0.00375 | 0.01125 | 0.06 | 0 | 0.00005 | 0 | 0.0776 |
| 48 | 40.4587 | 20.48548 | 0.042 | 0.105 | 0.105 | 0.0525 | 0.0042 | 0.0525 | 0.1575 | 0.168 | 0.0525 | 0.0525 | 0.0525 | 0.8442 |
| 49 | 40.45733 | 20.48559 | 0 | 0 | 0.0005 | 0.00175 | 0.0003 | 0.00375 | 0.01125 | 0.06 | 0 | 0.00005 | 0 | 0.0776 |
| 50 | 40.45955 | 20.48562 | 0 | 0 | 0.0005 | 0.00175 | 0.0003 | 0.00375 | 0.01125 | 0.06 | 0 | 0.00005 | 0 | 0.0776 |
| 51 | 40.457 | 20.4856 | 0 | 0 | 0.0005 | 0.00175 | 0.0003 | 0.00375 | 0.01125 | 0.06 | 0 | 0.00005 | 0 | 0.0776 |
| 52 | 40.45779 | 20.48573 | 0.0384 | 0.096 | 0.096 | 0.048 | 0.00384 | 0.048 | 0.144 | 0.1536 | 0.048 | 0.048 | 0.048 | 0.77184 |
| 53 | 40.45918 | 20.48574 | 0 | 0 | 0.0005 | 0.00175 | 0.0003 | 0.00375 | 0.01125 | 0.06 | 0 | 0.00005 | 0 | 0.0776 |
| 54 | 40.45823 | 20.48576 | 0.0486 | 0.1215 | 0.1215 | 0.06075 | 0.00486 | 0.06075 | 0.18225 | 0.1944 | 0.06075 | 0.06075 | 0.06075 | 0.97686 |
| 55 | 40.45879 | 20.48595 | 0.0192 | 0.048 | 0.048 | 0.024 | 0.00192 | 0.024 | 0.072 | 0.0768 | 0.024 | 0.024 | 0.024 | 0.38592 |
| 56 | 40.45909 | 20.48598 | 0.0185 | 0.04625 | 0.04625 | 0.023125 | 0.00185 | 0.023125 | 0.069375 | 0.074 | 0.023125 | 0.023125 | 0.023125 | 0.37185 |
| 57 | 40.45782 | 20.486 | 0.03 | 0.075 | 0.075 | 0.0375 | 0.003 | 0.0375 | 0.1125 | 0.12 | 0.0375 | 0.0375 | 0.0375 | 0.603 |
| 58 | 40.45715 | 20.48602 | 0 | 0 | 0.0005 | 0.00175 | 0.0003 | 0.00375 | 0.01125 | 0.06 | 0 | 0.00005 | 0 | 0.0776 |
| 59 | 40.45941 | 20.48607 | 0.0192 | 0.048 | 0.048 | 0.024 | 0.00192 | 0.024 | 0.072 | 0.0768 | 0.024 | 0.024 | 0.024 | 0.38592 |
| 60 | 40.45819 | 20.48612 | 0.03 | 0.075 | 0.075 | 0.0375 | 0.003 | 0.0375 | 0.1125 | 0.12 | 0.0375 | 0.0375 | 0.0375 | 0.603 |
| 61 | 40.45848 | 20.48617 | 0.03 | 0.075 | 0.075 | 0.0375 | 0.003 | 0.0375 | 0.1125 | 0.12 | 0.0375 | 0.0375 | 0.0375 | 0.603 |
| 62 | 40.459 | 20.48625 | 0.0185 | 0.04625 | 0.04625 | 0.023125 | 0.00185 | 0.023125 | 0.069375 | 0.074 | 0.023125 | 0.023125 | 0.023125 | 0.37185 |
| 63 | 40.45781 | 20.48626 | 0.03 | 0.075 | 0.075 | 0.0375 | 0.003 | 0.0375 | 0.1125 | 0.12 | 0.0375 | 0.0375 | 0.0375 | 0.603 |
| 64 | 40.45869 | 20.48634 | 0.032 | 0.08 | 0.08 | 0.04 | 0.0032 | 0.04 | 0.12 | 0.128 | 0.04 | 0.04 | 0.04 | 0.6432 |
| 65 | 40.45809 | 20.48643 | 0.032 | 0.08 | 0.08 | 0.04 | 0.0032 | 0.04 | 0.12 | 0.128 | 0.04 | 0.04 | 0.04 | 0.6432 |
| 66 | 40.45846 | 20.4865 | 0.032 | 0.08 | 0.08 | 0.04 | 0.0032 | 0.04 | 0.12 | 0.128 | 0.04 | 0.04 | 0.04 | 0.6432 |
| 67 | 40.45898 | 20.48652 | 0.032 | 0.08 | 0.08 | 0.04 | 0.0032 | 0.04 | 0.12 | 0.128 | 0.04 | 0.04 | 0.04 | 0.6432 |
| 68 | 40.45932 | 20.48651 | 0.027 | 0.0675 | 0.0675 | 0.03375 | 0.0027 | 0.03375 | 0.10125 | 0.108 | 0.03375 | 0.03375 | 0.03375 | 0.5427 |
| 69 | 40.45723 | 20.4866 | 0 | 0 | 0.0005 | 0.00175 | 0.0003 | 0.00375 | 0.01125 | 0.06 | 0 | 0.00005 | 0 | 0.0776 |
| 70 | 40.45791 | 20.48675 | 0.0384 | 0.096 | 0.096 | 0.048 | 0.00384 | 0.048 | 0.144 | 0.1536 | 0.048 | 0.048 | 0.048 | 0.77184 |
| 71 | 40.45732 | 20.48703 | 0 | 0 | 0.0005 | 0.00175 | 0.0003 | 0.00375 | 0.01125 | 0.06 | 0 | 0.00005 | 0 | 0.0776 |
| 72 | 40.45705 | 20.48738 | 0.042 | 0.105 | 0.105 | 0.0525 | 0.0042 | 0.0525 | 0.1575 | 0.168 | 0.0525 | 0.0525 | 0.0525 | 0.8442 |
| 73 | 40.45764 | 20.48921 | 0.039 | 0.0975 | 0.0975 | 0.04875 | 0.0039 | 0.04875 | 0.14625 | 0.156 | 0.04875 | 0.04875 | 0.04875 | 0.7839 |
| 74 | 40.45776 | 20.49098 | 0.0494 | 0.1235 | 0.1235 | 0.06175 | 0.00494 | 0.06175 | 0.18525 | 0.1976 | 0.06175 | 0.06175 | 0.06175 | 0.99294 |
| 75 | 40.45796 | 20.49287 | 0.042 | 0.105 | 0.105 | 0.0525 | 0.0042 | 0.0525 | 0.1575 | 0.168 | 0.0525 | 0.0525 | 0.0525 | 0.8442 |
| Average of Region 1 | | | 0.018348 | 0.04587 | 0.046097 | 0.023728 | 0.001971 | 0.024635 | 0.073905 | 0.100592 | 0.022935 | 0.022958 | 0.022935 | 40% |

Table S4. The parameters used as a critical aspect for making the geoelectrical model of sublayers depending on its related factors (Second region).

| Sounding No. | E | N | Weight Vector | | | | | | | | | | | Hypothetical Score (Geoelectrical Model of Sublayers for Suitability parameter) |
|---|---|---|---|---|---|---|---|---|---|---|---|---|---|---|
| | | | Resistivity (ρ) of Water Bearing Layer | Thickness (h) of Water Bearing Layer | Geometry of Water Bearing Layer | Boundaries of Water Bearing Layer | Electrode Configuration | Data Quality and Error Estimation | Inversion Algorithm | Geological Constraints | hydraulic conductivity | porosity | groundwater flow properties) | |
| 76 | 40.45823 | 20.49461 | 0.0375 | 0.1 | 0.1 | 0.05625 | 0.0075 | 0.03125 | 0.09375 | 0.225 | 0.03125 | 0.03125 | 0.03125 | 0.745 |
| 77 | 40.45885 | 20.49636 | 0.046286 | 0.123429 | 0.123429 | 0.069429 | 0.009257 | 0.038571 | 0.115714 | 0.277714 | 0.038571 | 0.038571 | 0.038571 | 0.919543 |
| 78 | 40.45984 | 20.49796 | 0.0495 | 0.132 | 0.132 | 0.07425 | 0.0099 | 0.04125 | 0.12375 | 0.297 | 0.04125 | 0.04125 | 0.04125 | 0.9834 |
| 79 | 40.46075 | 20.49951 | 0.044786 | 0.119429 | 0.119429 | 0.067179 | 0.008957 | 0.037321 | 0.111964 | 0.268714 | 0.037321 | 0.037321 | 0.037321 | 0.889743 |
| 80 | 40.46178 | 20.50106 | 0.045 | 0.12 | 0.12 | 0.0675 | 0.009 | 0.0375 | 0.1125 | 0.27 | 0.0375 | 0.0375 | 0.0375 | 0.894 |
| 81 | 40.46262 | 20.5027 | 0.045 | 0.12 | 0.12 | 0.0675 | 0.009 | 0.0375 | 0.1125 | 0.27 | 0.0375 | 0.0375 | 0.0375 | 0.894 |
| 82 | 40.46365 | 20.50421 | 0.044786 | 0.119429 | 0.119429 | 0.067179 | 0.008957 | 0.037321 | 0.111964 | 0.268714 | 0.037321 | 0.037321 | 0.037321 | 0.889743 |
| 83 | 40.46497 | 20.50576 | 0.045 | 0.12 | 0.12 | 0.0675 | 0.009 | 0.0375 | 0.1125 | 0.27 | 0.0375 | 0.0375 | 0.0375 | 0.894 |
| 84 | 40.46059 | 20.4743 | 0.0375 | 0.1 | 0.1 | 0.05625 | 0.0075 | 0.03125 | 0.09375 | 0.225 | 0.03125 | 0.03125 | 0.03125 | 0.745 |
| 85 | 40.46231 | 20.4751 | 0.013714 | 0.036571 | 0.036571 | 0.020571 | 0.002743 | 0.011429 | 0.034286 | 0.082286 | 0.011429 | 0.011429 | 0.011429 | 0.272457 |
| 86 | 40.46473 | 20.47575 | 0.013714 | 0.036571 | 0.036571 | 0.020571 | 0.002743 | 0.011429 | 0.034286 | 0.082286 | 0.011429 | 0.011429 | 0.011429 | 0.272457 |
| 87 | 40.46137 | 20.47257 | 0.013714 | 0.036571 | 0.036571 | 0.020571 | 0.002743 | 0.011429 | 0.034286 | 0.082286 | 0.011429 | 0.011429 | 0.011429 | 0.272457 |
| 88 | 40.46247 | 20.47307 | 0.013714 | 0.036571 | 0.036571 | 0.020571 | 0.002743 | 0.011429 | 0.034286 | 0.082286 | 0.011429 | 0.011429 | 0.011429 | 0.272457 |
| 89 | 40.4638 | 20.47336 | 0.013714 | 0.036571 | 0.036571 | 0.020571 | 0.002743 | 0.011429 | 0.034286 | 0.082286 | 0.011429 | 0.011429 | 0.011429 | 0.272457 |
| 90 | 40.46606 | 20.47358 | 0.029571 | 0.078857 | 0.078857 | 0.044357 | 0.005914 | 0.024643 | 0.073929 | 0.177429 | 0.024643 | 0.024643 | 0.024643 | 0.587486 |
| 91 | 40.4627 | 20.4704 | 0.013714 | 0.036571 | 0.036571 | 0.020571 | 0.002743 | 0.011429 | 0.034286 | 0.082286 | 0.011429 | 0.011429 | 0.011429 | 0.272457 |
| 92 | 40.46419 | 20.47018 | 0.014143 | 0.037714 | 0.037714 | 0.021214 | 0.002829 | 0.011786 | 0.035357 | 0.084857 | 0.011786 | 0.011786 | 0.011786 | 0.280971 |
| 93 | 40.46497 | 20.47025 | 0.028929 | 0.077143 | 0.077143 | 0.043393 | 0.005786 | 0.024107 | 0.072321 | 0.173571 | 0.024107 | 0.024107 | 0.024107 | 0.574714 |
| 94 | 40.46724 | 20.47018 | 0.014143 | 0.037714 | 0.037714 | 0.021214 | 0.002829 | 0.011786 | 0.035357 | 0.084857 | 0.011786 | 0.011786 | 0.011786 | 0.280971 |
| 95 | 40.4627 | 20.46837 | 0.014143 | 0.037714 | 0.037714 | 0.021214 | 0.002829 | 0.011786 | 0.035357 | 0.084857 | 0.011786 | 0.011786 | 0.011786 | 0.280971 |
| 96 | 40.46403 | 20.46772 | 0.040071 | 0.106857 | 0.106857 | 0.060107 | 0.008014 | 0.033393 | 0.100179 | 0.240429 | 0.033393 | 0.033393 | 0.033393 | 0.796086 |
| 97 | 40.46552 | 20.46743 | 0.0375 | 0.1 | 0.1 | 0.05625 | 0.0075 | 0.03125 | 0.09375 | 0.225 | 0.03125 | 0.03125 | 0.03125 | 0.745 |
| 98 | 40.46763 | 20.46844 | 0.038571 | 0.102857 | 0.102857 | 0.057857 | 0.007714 | 0.032143 | 0.096429 | 0.231429 | 0.032143 | 0.032143 | 0.032143 | 0.766286 |
| 99 | 40.46325 | 20.46641 | 0.040071 | 0.106857 | 0.106857 | 0.060107 | 0.008014 | 0.033393 | 0.100179 | 0.240429 | 0.033393 | 0.033393 | 0.033393 | 0.796086 |

| | | | | | | | | | | | | | | |
|---|---|---|---|---|---|---|---|---|---|---|---|---|---|---|
| 100 | 40.46473 | 20.46518 | 0.038571 | 0.102857 | 0.102857 | 0.057857 | 0.007714 | 0.032143 | 0.096429 | 0.231429 | 0.032143 | 0.032143 | 0.032143 | 0.766286 |
| 101 | 40.46653 | 20.46511 | 0.043714 | 0.116571 | 0.116571 | 0.065571 | 0.008743 | 0.036429 | 0.109286 | 0.262286 | 0.036429 | 0.036429 | 0.036429 | 0.868457 |
| 102 | 40.46864 | 20.46555 | 0.043714 | 0.116571 | 0.116571 | 0.065571 | 0.008743 | 0.036429 | 0.109286 | 0.262286 | 0.036429 | 0.036429 | 0.036429 | 0.868457 |
| 103 | 40.46364 | 20.46366 | 0.043714 | 0.116571 | 0.116571 | 0.065571 | 0.008743 | 0.036429 | 0.109286 | 0.262286 | 0.036429 | 0.036429 | 0.036429 | 0.868457 |
| 104 | 40.46684 | 20.46279 | 0.014143 | 0.037714 | 0.037714 | 0.021214 | 0.002829 | 0.011786 | 0.035357 | 0.084857 | 0.011786 | 0.011786 | 0.011786 | 0.280971 |
| 105 | 40.46942 | 20.46345 | 0.014571 | 0.038857 | 0.038857 | 0.021857 | 0.002914 | 0.012143 | 0.036429 | 0.087429 | 0.012143 | 0.012143 | 0.012143 | 0.289486 |
| 106 | 40.46395 | 20.46149 | 0.040071 | 0.106857 | 0.106857 | 0.060107 | 0.008014 | 0.033393 | 0.100179 | 0.240429 | 0.033393 | 0.033393 | 0.033393 | 0.796086 |
| 107 | 40.46591 | 20.46149 | 0.014571 | 0.038857 | 0.038857 | 0.021857 | 0.002914 | 0.012143 | 0.036429 | 0.087429 | 0.012143 | 0.012143 | 0.012143 | 0.289486 |
| 108 | 40.46778 | 20.46193 | 0.015 | 0.04 | 0.04 | 0.0225 | 0.003 | 0.0125 | 0.0375 | 0.09 | 0.0125 | 0.0125 | 0.0125 | 0.298 |
| 109 | 40.46989 | 20.46229 | 0.041529 | 0.110743 | 0.110743 | 0.062293 | 0.008306 | 0.034607 | 0.103821 | 0.249171 | 0.034607 | 0.034607 | 0.034607 | 0.825034 |
| 110 | 40.46591 | 20.45946 | 0.015 | 0.04 | 0.04 | 0.0225 | 0.003 | 0.0125 | 0.0375 | 0.09 | 0.0125 | 0.0125 | 0.0125 | 0.298 |
| 111 | 40.46724 | 20.45939 | 0.015 | 0.04 | 0.04 | 0.0225 | 0.003 | 0.0125 | 0.0375 | 0.09 | 0.0125 | 0.0125 | 0.0125 | 0.298 |
| 112 | 40.46802 | 20.45975 | 0.038571 | 0.102857 | 0.102857 | 0.057857 | 0.007714 | 0.032143 | 0.096429 | 0.231429 | 0.032143 | 0.032143 | 0.032143 | 0.766286 |
| 113 | 40.47005 | 20.45983 | 0.028929 | 0.077143 | 0.077143 | 0.043393 | 0.005786 | 0.024107 | 0.072321 | 0.173571 | 0.024107 | 0.024107 | 0.024107 | 0.574714 |
| 114 | 40.46606 | 20.45751 | 0.015 | 0.04 | 0.04 | 0.0225 | 0.003 | 0.0125 | 0.0375 | 0.09 | 0.0125 | 0.0125 | 0.0125 | 0.298 |
| 115 | 40.46786 | 20.45751 | 0.015 | 0.04 | 0.04 | 0.0225 | 0.003 | 0.0125 | 0.0375 | 0.09 | 0.0125 | 0.0125 | 0.0125 | 0.298 |
| 116 | 40.47036 | 20.45794 | 0.015 | 0.04 | 0.04 | 0.0225 | 0.003 | 0.0125 | 0.0375 | 0.09 | 0.0125 | 0.0125 | 0.0125 | 0.298 |
| 117 | 40.46458 | 20.45585 | 0.015 | 0.04 | 0.04 | 0.0225 | 0.003 | 0.0125 | 0.0375 | 0.09 | 0.0125 | 0.0125 | 0.0125 | 0.298 |
| 118 | 40.46606 | 20.45577 | 0.028929 | 0.077143 | 0.077143 | 0.043393 | 0.005786 | 0.024107 | 0.072321 | 0.173571 | 0.024107 | 0.024107 | 0.024107 | 0.574714 |
| 119 | 40.46724 | 20.45577 | 0.015 | 0.04 | 0.04 | 0.0225 | 0.003 | 0.0125 | 0.0375 | 0.09 | 0.0125 | 0.0125 | 0.0125 | 0.298 |
| 120 | 40.46849 | 20.45614 | 0.015 | 0.04 | 0.04 | 0.0225 | 0.003 | 0.0125 | 0.0375 | 0.09 | 0.0125 | 0.0125 | 0.0125 | 0.298 |
| 121 | 40.47075 | 20.45635 | 0.017143 | 0.045714 | 0.045714 | 0.025714 | 0.003429 | 0.014286 | 0.042857 | 0.102857 | 0.014286 | 0.014286 | 0.014286 | 0.340571 |
| 122 | 40.46575 | 20.4544 | 0.017143 | 0.045714 | 0.045714 | 0.025714 | 0.003429 | 0.014286 | 0.042857 | 0.102857 | 0.014286 | 0.014286 | 0.014286 | 0.340571 |
| 123 | 40.46708 | 20.45447 | 0.038571 | 0.102857 | 0.102857 | 0.057857 | 0.007714 | 0.032143 | 0.096429 | 0.231429 | 0.032143 | 0.032143 | 0.032143 | 0.766286 |
| 124 | 40.46903 | 20.45483 | 0.017143 | 0.045714 | 0.045714 | 0.025714 | 0.003429 | 0.014286 | 0.042857 | 0.102857 | 0.014286 | 0.014286 | 0.014286 | 0.340571 |
| 125 | 40.47091 | 20.45519 | 0.025714 | 0.068571 | 0.068571 | 0.038571 | 0.005143 | 0.021429 | 0.064286 | 0.154286 | 0.021429 | 0.021429 | 0.021429 | 0.510857 |
| 126 | 40.4663 | 20.45281 | 0.043714 | 0.116571 | 0.116571 | 0.065571 | 0.008743 | 0.036429 | 0.109286 | 0.262286 | 0.036429 | 0.036429 | 0.036429 | 0.868457 |
| 127 | 40.46794 | 20.45266 | 0.038571 | 0.102857 | 0.102857 | 0.057857 | 0.007714 | 0.032143 | 0.096429 | 0.231429 | 0.032143 | 0.032143 | 0.032143 | 0.766286 |
| 128 | 40.46919 | 20.45266 | 0.025714 | 0.068571 | 0.068571 | 0.038571 | 0.005143 | 0.021429 | 0.064286 | 0.154286 | 0.021429 | 0.021429 | 0.021429 | 0.510857 |
| 129 | 40.47122 | 20.45252 | 0.038571 | 0.102857 | 0.102857 | 0.057857 | 0.007714 | 0.032143 | 0.096429 | 0.231429 | 0.032143 | 0.032143 | 0.032143 | 0.766286 |
| 130 | 40.46786 | 20.45107 | 0.025714 | 0.068571 | 0.068571 | 0.038571 | 0.005143 | 0.021429 | 0.064286 | 0.154286 | 0.021429 | 0.021429 | 0.021429 | 0.510857 |
| 131 | 40.46966 | 20.45056 | 0.040071 | 0.106857 | 0.106857 | 0.060107 | 0.008014 | 0.033393 | 0.100179 | 0.240429 | 0.033393 | 0.033393 | 0.033393 | 0.796086 |
| 132 | 40.47177 | 20.45056 | 0.025714 | 0.068571 | 0.068571 | 0.038571 | 0.005143 | 0.021429 | 0.064286 | 0.154286 | 0.021429 | 0.021429 | 0.021429 | 0.510857 |
| 133 | 40.46802 | 20.44897 | 0.025714 | 0.068571 | 0.068571 | 0.038571 | 0.005143 | 0.021429 | 0.064286 | 0.154286 | 0.021429 | 0.021429 | 0.021429 | 0.510857 |

| | | | | | | | | | | | | | | |
|---|---|---|---|---|---|---|---|---|---|---|---|---|---|---|
| 134 | 40.46974 | 20.44875 | 0.025714 | 0.068571 | 0.068571 | 0.038571 | 0.005143 | 0.021429 | 0.064286 | 0.154286 | 0.021429 | 0.021429 | 0.021429 | 0.510857 |
| 135 | 40.47169 | 20.44875 | 0.040114 | 0.106971 | 0.106971 | 0.060171 | 0.008023 | 0.033429 | 0.100286 | 0.240686 | 0.033429 | 0.033429 | 0.033429 | 0.796937 |
| 136 | 40.47005 | 20.44672 | 0.025714 | 0.068571 | 0.068571 | 0.038571 | 0.005143 | 0.021429 | 0.064286 | 0.154286 | 0.021429 | 0.021429 | 0.021429 | 0.510857 |
| 137 | 40.47185 | 20.44607 | 0.042257 | 0.112686 | 0.112686 | 0.063386 | 0.008451 | 0.035214 | 0.105643 | 0.253543 | 0.035214 | 0.035214 | 0.035214 | 0.839509 |
| 138 | 40.47044 | 20.44499 | 0.0345 | 0.092 | 0.092 | 0.05175 | 0.0069 | 0.02875 | 0.08625 | 0.207 | 0.02875 | 0.02875 | 0.02875 | 0.6854 |
| 139 | 40.46935 | 20.44332 | 0.028929 | 0.077143 | 0.077143 | 0.043393 | 0.005786 | 0.024107 | 0.072321 | 0.173571 | 0.024107 | 0.024107 | 0.024107 | 0.574714 |
| 140 | 40.46763 | 20.44245 | 0.039643 | 0.105714 | 0.105714 | 0.059464 | 0.007929 | 0.033036 | 0.099107 | 0.237857 | 0.033036 | 0.033036 | 0.033036 | 0.787571 |
| 141 | 40.4695 | 20.44216 | 0.025714 | 0.068571 | 0.068571 | 0.038571 | 0.005143 | 0.021429 | 0.064286 | 0.154286 | 0.021429 | 0.021429 | 0.021429 | 0.510857 |
| 142 | 40.47005 | 20.43985 | 0.0345 | 0.092 | 0.092 | 0.05175 | 0.0069 | 0.02875 | 0.08625 | 0.207 | 0.02875 | 0.02875 | 0.02875 | 0.6854 |
| 143 | 40.4724 | 20.44166 | 0.039643 | 0.105714 | 0.105714 | 0.059464 | 0.007929 | 0.033036 | 0.099107 | 0.237857 | 0.033036 | 0.033036 | 0.033036 | 0.787571 |
| 144 | 40.47232 | 20.43941 | 0.039643 | 0.105714 | 0.105714 | 0.059464 | 0.007929 | 0.033036 | 0.099107 | 0.237857 | 0.033036 | 0.033036 | 0.033036 | 0.787571 |
| 145 | 40.46872 | 20.43608 | 0.028929 | 0.077143 | 0.077143 | 0.043393 | 0.005786 | 0.024107 | 0.072321 | 0.173571 | 0.024107 | 0.024107 | 0.024107 | 0.574714 |
| 146 | 40.47013 | 20.43789 | 0.039643 | 0.105714 | 0.105714 | 0.059464 | 0.007929 | 0.033036 | 0.099107 | 0.237857 | 0.033036 | 0.033036 | 0.033036 | 0.787571 |
| 147 | 40.47247 | 20.43811 | 0.039643 | 0.105714 | 0.105714 | 0.059464 | 0.007929 | 0.033036 | 0.099107 | 0.237857 | 0.033036 | 0.033036 | 0.033036 | 0.787571 |
| 148 | 40.46716 | 20.43239 | 0.039643 | 0.105714 | 0.105714 | 0.059464 | 0.007929 | 0.033036 | 0.099107 | 0.237857 | 0.033036 | 0.033036 | 0.033036 | 0.787571 |
| 149 | 40.46598 | 20.43094 | 0.039643 | 0.105714 | 0.105714 | 0.059464 | 0.007929 | 0.033036 | 0.099107 | 0.237857 | 0.033036 | 0.033036 | 0.033036 | 0.787571 |
| 150 | 40.46716 | 20.43008 | 0.039643 | 0.105714 | 0.105714 | 0.059464 | 0.007929 | 0.033036 | 0.099107 | 0.237857 | 0.033036 | 0.033036 | 0.033036 | 0.787571 |
| 151 | 40.47099 | 20.43369 | 0.03675 | 0.098 | 0.098 | 0.055125 | 0.00735 | 0.030625 | 0.091875 | 0.2205 | 0.030625 | 0.030625 | 0.030625 | 0.7301 |
| 152 | 40.4724 | 20.43623 | 0.025714 | 0.068571 | 0.068571 | 0.038571 | 0.005143 | 0.021429 | 0.064286 | 0.154286 | 0.021429 | 0.021429 | 0.021429 | 0.510857 |
| 153 | 40.46395 | 20.42971 | 0.040114 | 0.106971 | 0.106971 | 0.060171 | 0.008023 | 0.033429 | 0.100286 | 0.240686 | 0.033429 | 0.033429 | 0.033429 | 0.796937 |
| 154 | 40.46348 | 20.42805 | 0.025714 | 0.068571 | 0.068571 | 0.038571 | 0.005143 | 0.021429 | 0.064286 | 0.154286 | 0.021429 | 0.021429 | 0.021429 | 0.510857 |
| 155 | 40.46489 | 20.4274 | 0.040071 | 0.106857 | 0.106857 | 0.060107 | 0.008014 | 0.033393 | 0.100179 | 0.240429 | 0.033393 | 0.033393 | 0.033393 | 0.796086 |
| 156 | 40.46638 | 20.42703 | 0.040886 | 0.109029 | 0.109029 | 0.061329 | 0.008177 | 0.034071 | 0.102214 | 0.245314 | 0.034071 | 0.034071 | 0.034071 | 0.812263 |
| 157 | 40.4627 | 20.4308 | 0.043714 | 0.116571 | 0.116571 | 0.065571 | 0.008743 | 0.036429 | 0.109286 | 0.262286 | 0.036429 | 0.036429 | 0.036429 | 0.868457 |
| Average of Region 2 | | | 0.030449 | 0.081197 | 0.081197 | 0.045673 | 0.00609 | 0.025374 | 0.076122 | 0.182692 | 0.025374 | 0.025374 | 0.025374 | 60% |
